# Cell Cycle Control by a Minimal Cdk Network

**DOI:** 10.1371/journal.pcbi.1004056

**Published:** 2015-02-06

**Authors:** Claude Gérard, John J. Tyson, Damien Coudreuse, Béla Novák

**Affiliations:** 1 Oxford Centre for Integrative Systems Biology, Department of Biochemistry, University of Oxford, Oxford, United Kingdom; 2 Department of Biological Sciences, Virginia Tech, Blacksburg, Virginia, United States of America; 3 Institut de Génétique et Développement de Rennes, CNRS UMR 6290, Rennes, France; University of Virginia, UNITED STATES

## Abstract

In present-day eukaryotes, the cell division cycle is controlled by a complex network of interacting proteins, including members of the cyclin and cyclin-dependent protein kinase (Cdk) families, and the Anaphase Promoting Complex (APC). Successful progression through the cell cycle depends on precise, temporally ordered regulation of the functions of these proteins. In light of this complexity, it is surprising that in fission yeast, a minimal Cdk network consisting of a single cyclin-Cdk fusion protein can control DNA synthesis and mitosis in a manner that is indistinguishable from wild type. To improve our understanding of the cell cycle regulatory network, we built and analysed a mathematical model of the molecular interactions controlling the G1/S and G2/M transitions in these minimal cells. The model accounts for all observed properties of yeast strains operating with the fusion protein. Importantly, coupling the model’s predictions with experimental analysis of alternative minimal cells, we uncover an explanation for the unexpected fact that elimination of inhibitory phosphorylation of Cdk is benign in these strains while it strongly affects normal cells. Furthermore, in the strain without inhibitory phosphorylation of the fusion protein, the distribution of cell size at division is unusually broad, an observation that is accounted for by stochastic simulations of the model. Our approach provides novel insights into the organization and quantitative regulation of wild type cell cycle progression. In particular, it leads us to propose a new mechanistic model for the phenomenon of mitotic catastrophe, relying on a combination of unregulated, multi-cyclin-dependent Cdk activities.

## Author Summary

The eukaryotic cell division cycle is driven by fluctuating activities of cyclin-dependent kinases (Cdk), which are activated and inactivated by several mechanisms, including cyclin synthesis and degradation. Although the cell cycle is driven by many different Cdk-cyclin complexes in present-day eukaryotes, experiments with fission yeast demonstrate that a single Cdk-cyclin complex is sufficient to order the events of the cell cycle. Surprisingly, a Cdk-inhibitory mechanism working through tyrosine phosphorylation of the kinase subunit, which is essential for modern fission yeast, becomes dispensable in the Minimal Cdk Network (MCN). By developing both deterministic and stochastic models of the MCN, we show that a different inhibitory mechanism based on a stoichiometric Cdk inhibitor (called Rum1) can compensate for the lack of inhibitory Cdk phosphorylation in the MCN. We also demonstrate that this compensation mechanism is suppressed in wild-type fission yeast cells by the other Cdk-cyclin complexes, which down-regulate the level of Rum1. These predictions of computational modelling are supported by our experimental data. Our work provides new insights into the interplay between the structure of the control network and the physiology of the cell cycle.

## Introduction

The cell division cycle plays a crucial role in the growth, development, repair and reproduction of living organisms in both normal and pathological conditions. Progression through the cell cycle requires faithful replication of the genome during S phase (DNA synthesis) and equal partitioning of the replicated chromosomes to the two daughter cells during mitosis and cell division (M phase). Because strict alternation of S and M phases is essential for successful cell proliferation, the mechanisms responsible for the temporal ordering of these two events are of fundamental importance to all eukaryotic cell life [1].

### Qualitative and quantitative control mechanisms

S and M are triggered by the phosphorylation of specific cellular proteins by a family of protein kinases, called cyclin-dependent kinases (Cdk’s) [2]. The activity of a Cdk depends on obligatory association with a regulatory subunit of the cyclin family, and a variety of Cdk:cyclin complexes are responsible for initiating DNA replication and mitosis in present-day eukaryotes. These observations naturally led to the “qualitative model” of cell cycle control, in which the temporal alternation of S and M is a consequence of alternating oscillations of at least two different Cdk:cyclin complexes, SPF (S-phase promoting factor) and MPF (M-phase promoting factor), with different substrate specificities [3].

This qualitative model might be true for cell cycle control in higher eukaryotes, but it is difficult to reconcile with the fact that a single Cdk1:cyclin B complex can drive an ordered sequence of S and M phases in fission yeast [4, 5]. (In fission yeast, Cdk1 is encoded by the *cdc2* gene and its only essential partner, a B-type cyclin, is encoded by *cdc13*.) The observation that Cdc2:Cdc13 alone is sufficient to orchestrate the fission yeast cell cycle led to a “quantitative model” of cell cycle control [4, 6], in which low Cdc2 activity is sufficient to trigger DNA replication while high activity blocks re-replication and brings about mitosis.

Strong experimental support for the quantitative model was provided by the demonstration that a Cdc13-Cdc2 fusion protein (called Cdc13-L-Cdc2, L for “linker”) can by itself drive cell cycle progression in a manner that is indistinguishable from wild type fission yeast cells [7]. In the minimal strain (genotype: *cdc13-L-cdc2 Δcdc13 Δcdc2*), the genomic copies of *cdc13* and *cdc2* have been deleted, so that cells cannot make normal Cdc2:Cdc13 heterodimers and therefore rely solely on the fusion protein for MPF activity. In addition, because these cells lack Cdc2 monomers, they should not be able to make heterodimers of Cdc2 with G1- or S-specific cyclins (Cig1, Cig2 and Puc1, encoded by *cig1*, *cig2*, and *puc1*, respectively). Nevertheless, to prevent the formation of potential alternative complexes between the Cdc2 moiety of the fusion protein and these cyclins (e.g. Cdc13-L-Cdc2:Cig2), which may contribute to temporal ordering of the cell cycle, the three other fission yeast cell cycle cyclin genes were deleted (*Δcig1 Δcig2 Δpuc1*, referred to as *ΔCCP*). Strikingly, *cdc13-L-cdc2 Δcdc13 Δcdc2 ΔCCP* cells progress through S and M in perfectly wild type fashion, indicating that the fusion protein Cdc13-L-Cdc2 has both SPF and MPF activities. We will refer to cell cycle control in this strain as the “Minimal Cdk Network”, and we will use *MCN* to denote the genotype of these cells (i.e., *cdc13-L-cdc2 Δcdc13 Δcdc2 ΔCCP*).

### Phenotypic characterization of MCN-derived strains

Both wild type and *MCN* cell cycles are characterized by a very short G1 phase (from the end of M phase to the onset of S phase) and a long G2 phase (from the end of S phase to the onset of mitosis); and both types of cells divide at a length of 14–16 μm (Table 1). The long duration of G2 results from inhibition of Cdc2:Cdc13 activity by the Wee1 and Mik1 kinases [8, 9]. However, loss-of-function of these inhibitory kinases has very different consequences in wild type and *MCN* cells. In wild type cells, inactivation of Wee1 (using a temperature-sensitive mutation, *wee1–50^ts^*) advances cells into mitosis, shortening the time spent in G2 and reducing cell size at division by almost 50% [10]. Furthermore, simultaneous inactivation of both Wee1 and Mik1 (*wee1–50^ts^ Δmik1* double mutant) or mutation of their target sites in Cdc2 into non-phosphorylable residues (T14A and Y15F, referred to as *cdc2AF*) drives otherwise wild type cells into mitosis before they have completed DNA synthesis, a lethal situation called “mitotic catastrophe” [9, 11, 12]. In contrast, *MCN* cells devoid of Cdc2 inhibitory phosphorylation (i.e., *MCN Δwee1 Δmik1* and *MCN-AF*) are perfectly viable, and their average size at division is comparable to that in wild type cells (Table 1). These unusual and intriguing properties of *MCN* cells have prompted us to develop a mathematical model of the minimal Cdk network that addresses, in particular, the central question of why Cdk1 inhibitory phosphorylation is mostly essential in wild type cells but dispensable in *MCN* cells.

**Table 1:** Observed phenotypes of fission yeast mutants discussed in this study.

Row	Genotype	Phenotype	Reference
**1**	Wild type	Short G1, long G2, DL = 14 μm	[10]
**2**	*wee1–50^ts^*	Long G1, short G2, DL = 6.9 μm	[10]
**3**	*wee1–50^ts^ Δmik1*	Inviable: mitotic catastrophe	[9]
**4**	*cdc2AF*	Inviable: mitotic catastrophe	[11]
**5**	*cdc13-L-cdc2 Δcdc13 Δcdc2*	Short G1, long G2, DL = 15.6 μm	[7]
**6**	*cdc13-L-cdc2 Δcdc13 Δcdc2 ΔCCP*	Short G1, long G2, DL = 15.9 μm	[7]
**7**	*cdc13-L-cdc2 Δcdc13 Δcdc2 ΔCCP Δrum1*	Short G1, long G2, DL = 15.9 μm	[7]
**8**	*cdc13-L-cdc2 Δcdc13 Δcdc2 ΔCCP Δwee1 Δmik1*	Long G1, short G2, DL = 13.7 μm, conditional mitotic catastrophe	[7]
**9**	*cdc13-L-cdc2-AF Δcdc13 Δcdc2 ΔCCP*	Long G1, short G2, DL = 13.9 μm, conditional mitotic catastrophe	[7]
**10**	*cdc13-L-cdc2 cdc2^+^Δcdc13 CCP^+^*	Short G1, long G2, DL = 10.5 μm	This study^*^
**11**	*cdc13-L-cdc2AF cdc2^+^Δcdc13 CCP* ^+^	Long G1, short G2, DL = 8.8 μm	This study^#^
**12**	*cdc13-L-cdc2AF Δcdc13 Δcdc2 ΔCCP Δrum1*	Inviable	This study
**13**	*cdc13-L-cdc2 Δcdc13 Δcdc2 ΔCCP Δwee1 Δmik1 Δrum1*	Inviable	This study
**14**	*ΔCCP*	Wild type^**^	This study
**15**	*wee1–50^ts^ Δmik1 ΔCCP*	Long G1 at permissive temperature Rescue of the *wee1–50^ts^ Δmik1* phenotype at restrictive temperature (5h—lower incidence of “cut”)	This study

DL = division length

^*^ generation time = 160 mins

^#^ generation time = 218 mins

^**^ DL non-determined

### Mathematical model of MCN cells

If, as experiments suggest, the fundamental timing of S and M phases in the fission yeast cell cycle is a quantitative property of the temporally varying activity of Cdk1, then the phenotypic properties of wild type and *MCN* cells cannot be accurately explained in terms of qualitative reasoning about genetic effects. Rather, coupling experimental results with a quantitative, computational model is necessary to capture the consequences of genetic manipulations in the context of wild type and *MCN* genetic backgrounds. By creating a mathematical model of the interactions of the fusion protein with Wee1 and other Cdk-regulatory proteins, we provide a consistent, quantitative understanding of how such a minimal control system can direct perfectly normal fission yeast cell cycles. Furthermore, confronting the model’s predictions with the phenotype of alternative minimal yeast cells, we propose an explanation for the paradoxical effects of abrogating inhibitory phosphorylation of Cdk1 in wild type and *MCN* backgrounds, thereby revisiting the mechanistic origin of mitotic catastrophe. Thus, not only does our model behave identically to the *MCN* strains described by Coudreuse & Nurse [7], but it also has important implications for wild type cell cycle control, shedding new light on the functional interactions between Cdk and its inhibitor Rum1, on the roles of CCP-dependent Cdk activity in regulating the timing of mitosis, and on the effects of molecular noise on cell cycle robustness.

## Results

### Temporal dynamics of the minimal Cdk network

**Figure 1:**
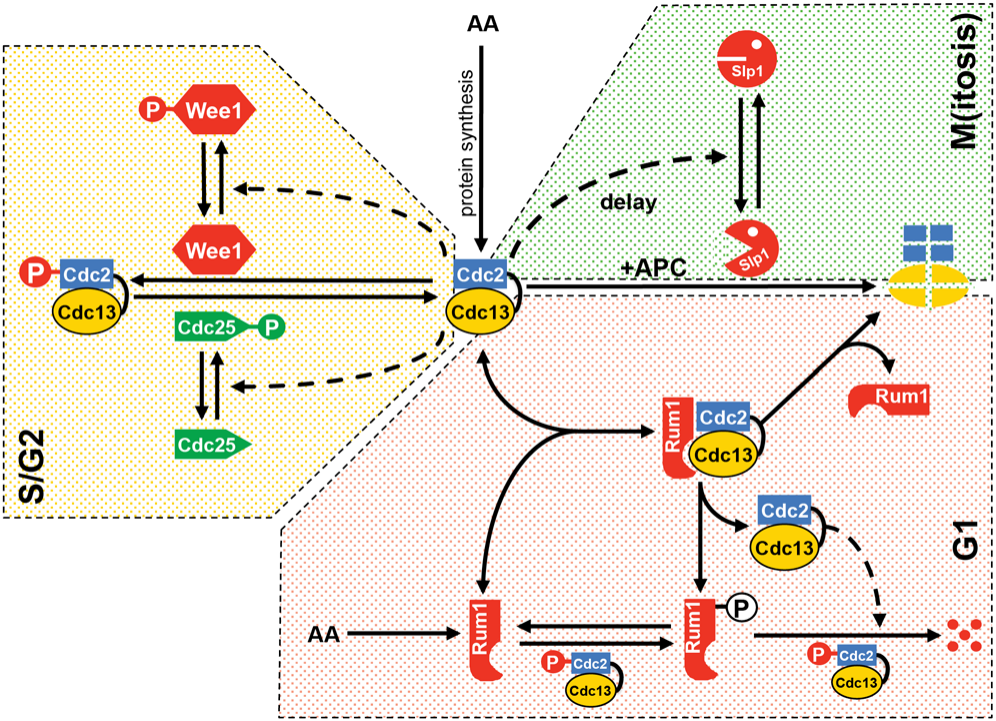
Reaction scheme for the minimal Cdk network driving the cell cycle in fission yeast. Solid lines represent biochemical reactions, while dashed lines define catalytic effects. Only one Cdk:cyclin complex (the fusion protein Cdc13-L-Cdc2, referred to as MPF) controls the successive progression through DNA replication and mitosis. MPF activity can be regulated by reversible association with the Cdk inhibitor Rum1, as well as by phosphorylation and dephosphorylation by the inhibitory kinase Wee1 and the activating phosphatase Cdc25, respectively. MPF inhibits Rum1 and Wee1, while it activates Cdc25. These regulatory interactions create mutual inhibitions between MPF and Rum1 and between MPF and Wee1, and a mutual activation loop between MPF and Cdc25. In the model, we consider that the phosphorylated form of MPF, MPF_P_, is still partially active (5% of the activity of MPF). Thus, MPF_P_ can inhibit Rum1 and regulate Wee1 and Cdc25. We assume that MPF and Rum1 form a stable complex, but the binding of Rum1 to MPF_P_ is much less strong. Active MPF promotes its own degradation through a delayed negative feedback loop involving Slp1 and the APC (Anaphase Promoting Complex). This negative feedback loop, which causes the destruction of MPF at the end of mitosis, is critical to generating sustained oscillations in MPF activity that drive repetitive cycles of DNA replication followed by mitosis. S1 Table defines the different proteins involved in the model. The “time delay” in the figure is implemented in the differential equations by an Intermediary Enzyme (IE) between MPF and Slp1.

The ‘Minimal Cdk Network’ presented in Fig. 1 was converted into a set of kinetic equations (S1 and S2 Tables) based on the assumptions described in the Methods section (Mathematical modelling). These non-linear ordinary differential equations were solved numerically for carefully chosen kinetic parameter values (S3 Table) to generate the time evolution of the variables of the minimal network.

**Figure 2:**
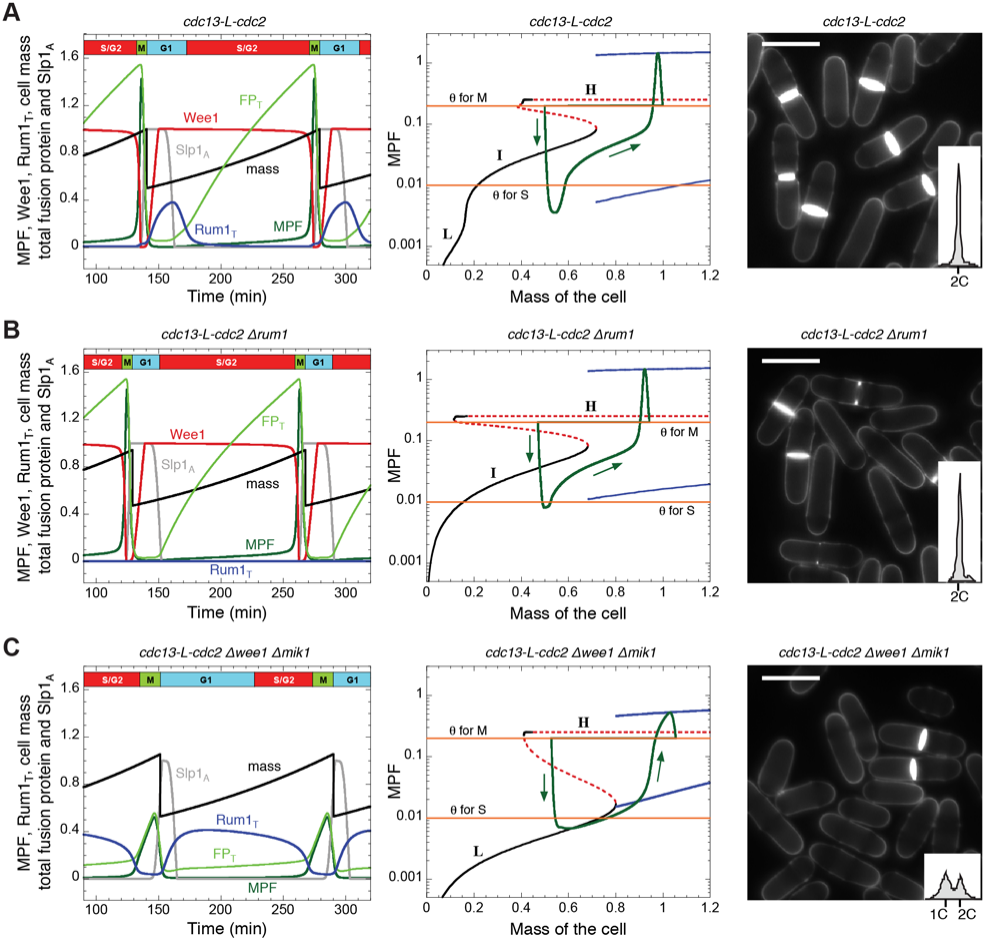
Dynamical behaviour of the minimal Cdk network, as defined by the differential equations in S2 Table and the parameter values in S3 Table, except where otherwise noted. **A.** *MCN* strain (*cdc13-L-cdc2 Δcdc13 Δcdc2 ΔCCP*). **B.** *MCN Δrum1* strain (*V*_SRUM1_ = 0). **C.** *MCN Δwee1 Δmik1* strain (*Wee1*_T_ = 0). In the left panels in A, B and C, we plot the time evolution of total fusion protein (light green, FP_T_), active MPF (dark green), active Wee1 (red), total Rum1 (blue), active APC:Slp1 (grey) and cell mass (black). Total Rum1 is defined by Rum1_T_ = Rum1 + Rum1_P_ + MPF:Rum1. The corresponding bifurcation diagrams for MPF as a function of cell mass are plotted in the middle panels. In these diagrams, black curves represent stable steady states, red dashed curves define unstable states, and blue curves indicate the envelope (i.e. maxima and minima) of the sustained oscillations. Superimposed on each bifurcation diagram is a projection of the cell cycle trajectory (green curve), *MPF*(*t*) versus *m*(*t*), as given by the time courses in the panel directly to its left. Arrows indicate the direction of movement of the system around the closed orbit. In the middle panels of A and B, we see that, for a brief G1 phase shortly after cell division, MPF activity is low, allowing cells to re-license the origins of DNA replication. The subsequent rise in MPF activity across the *θ_S_* threshold defines entry into S phase. The phase of DNA synthesis is quite brief in fission yeast cells (20–30 min), after which the cell is in G2 phase, by definition. When the cell grows large enough, it generates an abrupt peak of MPF activity, which drives the cell into M phase (when *MPF* crosses the threshold *θ*_M_). Subsequently, MPF activity is inhibited through degradation of the cyclin (or fusion protein in *MCN* cells) by APC:Slp1. When *MPF* drops below *θ*_M_, the cell divides. The case for *MCN Δwee1 Δmik1* cells, middle panel in C, is considerably different. The model predicts that an elevated level of Rum1 in this strain is responsible for an extended duration of G1 phase. Nonetheless, cell mass at division is similar in all three strains, consistent with experimental data [7]. Right panels show blankophor staining of exponentially growing cells in the three conditions together with flow cytometry analysis of their DNA content. Scale bar = 10 μm. All strains carry deletions of the endogenous copies of *cdc2*, *cdc13*, *cig1*, *cig2* and *puc1*.

In Fig. 2A (left panel), we plot the time evolution of some representative variables in our mathematical model of *MCN* cells: the active forms of MPF and Wee1, the total levels of Rum1 (Rum1_T_) and Cdc13-L-Cdc2 (FP_T_), the activity of the Anaphase Promoting Complex (APC:Slp1) and cell mass as a proxy for cell size. Between successive cell divisions, we can distinguish three stages of MPF activity. During the initial, brief stage immediately after cell division, MPF activity is close to zero as a result of Rum1-dependent inhibition and degradation of MPF. Low MPF activity allows for the re-accumulation of replication licensing factors (Cdc18 and Cdt1) at the replication origins in the yeast genome [13]. During the following intermediate stage, the total amount of fusion protein rises quickly, but MPF activity rises slowly as a consequence of Wee1-mediated conversion of MPF to its phosphorylated, inhibited form, MPF_P_. Finally, a transient stage of high MPF activity is observed during mitosis, as MPF_P_ is abruptly converted to MPF by the Cdc25 phosphatase. Note that inhibitory phosphorylation of Cdc2 is the rate-limiting process for cell cycle progression in *MCN* cells, as it is also in wild type cells.

In both experiments and simulations, loss of Rum1 from the minimal Cdk network (strain *cdc13-L-cdc2 Δcdc13 Δcdc2 ΔCCP Δrum1 = MCN Δrum1*) introduces no obvious changes in the temporal pattern of MPF activity and cell size (compare left and right panels of Fig. 2A with Fig. 2B; see also Table 1 Row 7 and Table 2 Row 2), because Rum1 is not rate-limiting for cell cycle progression in this background. MPF activity is still characterized by three stages with low, intermediate and high activities. The low activity of MPF in early G1 phase in this simulation is a consequence solely of APC:Slp1-dependent degradation of the fusion protein.

**Table 2:** Computed phenotypes of fission yeast mutants discussed in this study.

**Row**	**Genotype**	**G1 (min)**	**S/G2 (min)**	**M (min)**	Re-licensing time^*^ (min)	**Mass at division**
**1**	*cdc13-L-cdc2 Δcdc13 Δcdc2 ΔCCP*	31.8	98.5	8.3	27.1	1.00
**2**	*cdc13-L-cdc2 Δcdc13 Δcdc2 ΔCCP Δrum1*	21.5	108.6	8.5	13.8	0.94
**3**	*cdc13-L-cdc2 Δcdc13 Δcdc2 ΔCCP Δwee1 Δmik1*	75.0	46.7	16.9	68.7	1.05
**4**	*cdc13-L-cdc2AF Δcdc13 Δcdc2 ΔCCP*	75.0	46.7	16.9	68.7	1.05
**5**	*cdc13-L-cdc2 Δcdc13 cdc2^+^ CCP* ^+^ (CCP = 1)	25.7	104.5	8.4	19.9	0.98
**6**	*cdc13-L-cdc2AF Δcdc13 cdc2^+^ CCP* ^+^ (CCP = 1)	50.3	63.1	25.2	42.3	0.59
**7**	*cdc13-L-cdc2 Δcdc13 cdc2AF CCP* ^+^ (CCP = 2)	23.5	106.7	8.4	16.9	0.97
**8**	*cdc13-L-cdc2AF Δcdc13 cdc2AF CCP* ^+^ (CCP = 2)	Not viable			0.0	0.41
**9**	*cdc13-L-cdc2AF Δcdc13 Δcdc2 ΔCCP Δrum1*	Not viable			0.0	0.18

^*^Re-licensing time = duration of the phase when MPF activity < *θ*_S_.

Removal of Wee1 from the minimal Cdk network (*MCN Δwee1 Δmik1* cells) is simulated in Fig. 2C, left panel. Although the lack of inhibitory phosphorylation changes the temporal dynamics of MPF activity, it does not significantly affect cell size at division (Fig. 2C right panel, and Table 1 Row 8), as observed experimentally [7]. However, the duration of the low MPF activity state (corresponding to G1) is extended, as reflected by the accumulation of cells with a 1C DNA content (Fig. 2C, right panel inset), due to the persistence of high levels of Rum1, whose degradation has become the rate-limiting step in cell cycle progression.

To understand the unusual phenotype of *MCN Δwee1 Δmik1* cells, as compared to *Δwee1 Δmik1* in a wild type background (see below), let us return to a consideration of *MCN* cells. In the presence of Wee1, the fusion protein in our model accumulates first in a phosphorylated form, MPF_P_, which is not efficiently inhibited by Rum1. We assume that MPF_P_ binds only weakly to Rum1, limiting the effect of persistent stoichiometric inhibition of MPF_P_ by Rum1 but allowing phosphorylation of Rum1 by MPF_P_. Hence, despite its lower intrinsic activity compared to MPF, MPF_P_ can promote early phosphorylation and subsequent degradation of Rum1. Contrariwise, in the absence of Wee1, the fusion protein accumulates in its unphosphorylated form, which is strongly inhibited by tight binding to Rum1. MPF-dependent mono-phosphorylation of Rum1 is therefore slow and Rum1_P_ is rapidly dephosphorylated by a phosphatase. Because MPF preferentially re-associates with non-phosphorylated Rum1, the rate of multi-phosphorylation of Rum1 and its subsequent degradation is strongly reduced, leading to the maintenance of high levels of Rum1. Therefore, the mutant cells (*MCN Δwee1 Δmik1*) must first accumulate enough fusion protein to titrate out non-phosphorylated Rum1; only then will there be some free MPF available to catalyse the second phosphorylation of Rum1_P_ (followed by subsequent degradation of Rum1_P2_), allowing entry into the next regime of MPF activity. (See Mathematical modelling in the Methods section for further details concerning the assumptions we have made about the binding of Rum1 to MPF and to MPF_P_ and the rates of phosphorylation of Rum1 by the two forms of MPF.)

The persistence of significant levels of active Rum1 in the *MCN Δwee1 Δmik1* cells provides a mechanistic explanation for the observed viability and extended G1 phase in this genetic background (see [7] and Fig. 2C right panel). If our understanding of the role of Rum1 in these cells is correct, then *rum1* should be an essential gene in *MCN Δwee1 Δmik1* cells, a prediction confirmed by our observation that deletion of *rum1* in the *MCN Δwee1 Δmik1* background is lethal (Table 1 Row 13; see also Experimental procedures section). Our model also predicts that the G2/M transition in this strain is brought about by abrupt Rum1 degradation rather than by Cdc2 dephosphorylation (as is the case in wild type and *MCN* cells).

In a wild type genetic background, *Δwee1 Δmik1* cells enter M phase prematurely and undergo unconditional “mitotic catastrophe”, i.e. they divide before they have completed DNA replication [14]. Hence, newborn cells do not receive complete copies of the genome and eventually die. In the *MCN* genetic background, *Δwee1 Δmik1* cells avoid mitotic catastrophe because they have a long gap (~50 min) between the onset of DNA synthesis and entry into mitosis resulting from MPF inhibition by Rum1. However, *MCN Δwee1 Δmik1* cells lack an active S phase checkpoint and hence remain subject to conditional mitotic catastrophe; i.e. if DNA synthesis is challenged by drugs such as hydroxyurea, these cells enter mitosis with incompletely replicated DNA and die [7]. Importantly, applying our mathematical approach to the particularities of these *MCN*-derived cells provides a novel opportunity to study the causes of mitotic catastrophe in fission yeast cells devoid of Cdc2 inhibitory phosphorylation, as will be described in a later paragraph.

### Bifurcation diagrams of the cell division cycle in *MCN* cells

It is instructive to analyse our model of *MCN* cell cycles on one-parameter bifurcation diagrams [15, 16]. A one-parameter bifurcation diagram plots the stable and unstable attractors (steady states and oscillations) of a dynamical system as functions of a “control parameter”. In the case of yeast cell cycle regulation, it is sensible to choose cell mass (*m*) as the control parameter, because cell growth (increase in *m*) is a major driving force for cell cycle progression in lower eukaryotes. In our model, *m* increases slowly and exponentially, so one may think of the bifurcation parameter as *e^μt^*, where *μ* is the specific growth rate of the cell in the culture medium. Hence, progression in time also corresponds to movement from left to right along the horizontal axis of the one-parameter bifurcation diagram.

By defining distinct MPF-thresholds for initiation of S and M phases (*θ*_S_ = 0.01 and *θ*_M_ = 0.2), MPF activity can be divided into three regimes (Fig. 2A, middle panel), which can be associated with G1, S/G2 and M activities. For MCN cells, the bifurcation diagram shows three distinct and partially overlapping steady states of MPF activity (Low, Intermediate and High) within these regimes. The low and intermediate steady states are stable, while the high steady states are unstable and surrounded by stable limit cycle oscillations. The low MPF activity steady state is defined by Rum1-dependent inhibition of MPF and degradation of Cdc13-L-Cdc2 by APC. The intermediate MPF activity state is stabilized by Wee1-dependent inhibition of MPF. The high activity state is destabilized by the negative feedback loop between MPF and APC:Slp1. Wherever a stable steady state ends with increasing cell mass (i.e., increasing time), the control network must jump to the next stable state, which corresponds to a cell cycle transition. For our choice of parameter values (S3 Table), the transition from L to I is smooth (not associated with a bifurcation), but the two steady states are still qualitatively different: in the L state, MPF is inhibited by high levels of Rum1; in the I state, MPF activity is downregulated by Wee1. In contrast, the transition from I to H is abrupt and defined by a SNIC bifurcation (“saddle-node on an invariant circle” [15]), corresponding to the transition from G2 phase (intermediate activity of MPF) to M phase (high activity of MPF).

By overlaying the time course of a cell cycle simulation (MPF activity and mass from Fig. 2A, left panel) on the bifurcation diagram, we plot how the “cell cycle states” in the model change during progression through a *MCN* cycle (green curve in the middle panel of Fig. 2A). To describe this cycle, we start where the green curve rises above *θ*_M_ = 0.2, the threshold for MPF initiation of mitosis. MPF activity increases rapidly, but it cannot settle on the H steady state because H is unstable. Shortly after the cell enters M phase, it exits mitosis as activated APC:Slp1 marks the fusion protein for degradation. When MPF activity drops below *θ*_M_, the cell divides and *m* is reset to *m*/2 (1.00 to 0.50). (For simplicity, we assume that the threshold for exit from mitosis is identical to the threshold for entry into mitosis.) The newborn cell enters the domain of attraction of the stable I steady state, but its trajectory on the way to this attractor carries it to a lower level of MPF activity (0.0035). It is necessary for MPF to drop to such low activity in order to allow the relicensing of the DNA replication origins. Subsequently, as the cell grows and MPF activity increases again (it is being attracted to the I steady state), the green curve crosses *θ*_S_ = 0.01, the threshold for MPF-triggered initiation of DNA synthesis. At this point, the young cell leaves the G1 phase of the cell cycle (unreplicated chromosomes) and enters S phase (replicating chromosomes). Note that the *MCN* cell never visits the L steady state of the control network, which corresponds to a stable G1-like phase. G1 only represents a short, transient phase of the cell cycle in fission yeast because the previously described G1/S size control is cryptic [17]. In addition, the cell trajectory (green curve) on the bifurcation diagram never gets very close to the I stable steady state, due to the slow turn-over of the fusion protein when APC is inactive. Therefore cell growth carries the cell past the SNIC bifurcation into the domain of attraction of the stable limit cycle oscillations, before it can reach the I attractor. Finally, MPF activity undergoes an explosive rise, which carries it across *θ*_M_, where we started this tour of the *MCN* cell cycle.

### Bifurcation diagrams of mutant *MCN* cell cycles

For Rum1-depleted MCN cells, the Wee1/Mik1-mediated activity state (I) is extended to a broader range, including the low MPF activity regime at very small cell size (Fig. 2B, middle panel). However, since the cell cycle trajectory of *MCN rum1* ^+^ cells never approaches the stable L state, *rum1* deletion has no discernible effect on cell cycle progression (compare middle panels of Figs. 2A and 2B), as observed (Table 1 Row 7). Because they lack the Rum1-dependent L state, Δ*rum1* cells cannot be arrested in G1 with mating pheromones [18].

Depletion of Wee1 and Mik1 from *MCN* cells eliminates the stable I state from the bifurcation diagram (Fig. 2C, middle panel), as expected. However, the Rum1-dependent L state now becomes extended over the MPF threshold for DNA replication (*θ*_S_) into the S/G2 activity regime and terminates at a SNIC bifurcation point at *m* ≈ 0.7, which is nearly the same size as the SNIC bifurcation in the background strain (*MCN*). Hence, *MCN Δwee1 Δmik1* cells divide at about the same size as *MCN* cells (see Tables 1 and 2). Compared to the initial *MCN* strain, the stable limit cycles in the absence of Wee1 and Mik1 have a smaller amplitude, but this is of little practical effect because cell division carries the cell cycle trajectory into the domain of attraction of the stable L state. Roughly half of the trajectory for the growing newborn cell lies close to the L attractor, consistent with the fact that these cells have an extended G1 phase (Fig. 2C right panel, Table 1 Row 8 and Table 2 Row 3). Note that DNA synthesis is initiated when the cell cycle trajectory (the green curve in the middle panel of Fig. 2C) crosses *θ*_S_, while the control system is still in the domain of attraction of the stable L steady state. For this reason, there is a significant time delay (~50 min) between the onset of DNA synthesis (when the green curve crosses *θ*_S_) and entry into mitosis (when the green curve crosses *θ*_M_), which prevents these cells from undergoing an unconditional mitotic catastrophe, despite the fact that they lack an effective S phase checkpoint.

### Conditional mitotic catastrophe

Coudreuse & Nurse [7] observed that lack of inhibitory Cdc2 phosphorylation in *MCN* cells surprisingly preserves both viability and average cell size at division, in contradiction to the properties of wild type fission yeast cells (Table 1). Inactivation (or deletion) of Wee1 in otherwise wild type cells reduces cell size at division by ~50%, and complete elimination of inhibitory Cdc2 phosphorylation (*wee1^ts^ mik1Δ*, *wee1^ts^ cdc25^op^*, or *cdc2AF*) strongly affects cell viability, with a high incidence of death by unconditional mitotic catastrophe. Why do similar mutations in the *MCN* background have such different effects?

Interestingly, Coudreuse & Nurse [7] also demonstrated that *MCN* cells can exhibit “conditional” mitotic catastrophe after “G1 reset”. To do this experiment, they introduced the Shokat mutation [19], *cdc2_as_*, into the fusion protein in the *MCN* strain, thereby making MPF activity responsive to the ATP analog NmPP1. The protocol of a “G1 reset” experiment is: (1) arrest cells in G2 with a low dose of inhibitor (1 μM NmPP1) so that cells complete DNA synthesis but do not enter mitosis; (2) transfer cells to a high dose of inhibitor (10 μM NmPP1) to reduce MPF activity to very low level, thereby re-setting cells to G1 without an intervening mitosis and re-licensing origins of DNA replication; (3) transfer cells to inhibitor-free medium to allow rapid rise in MPF activity. Under these conditions, cells initiate a new round of DNA synthesis, but also rapidly enter into mitosis and show a “cut” phenotype, indicating that they are dividing with incompletely replicated chromosomes.

**Figure 3:**
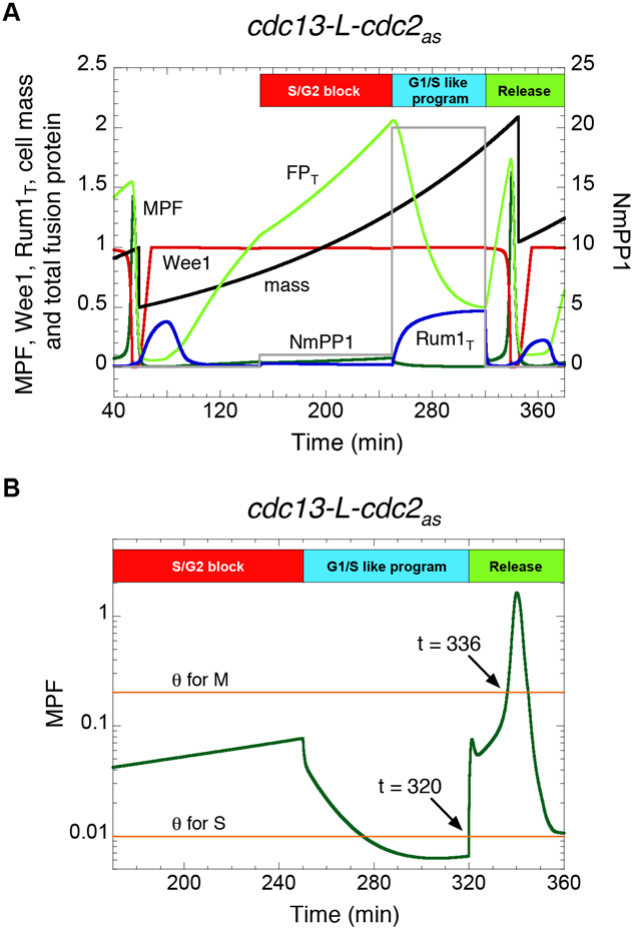
Conditional mitotic catastrophe in *MCN* cells carrying the Shokat mutation in the Cdc2 moiety of the fusion protein. To model the effects of the Shokat inhibitor, NmPP1 [19], on this strain, we replace the variable *MPF* in S2 Table by *MPF*/(1+*NmPP1*), where *NmPP1* = [NmPP1]/IC50. **A.** Simulation of the “G1-reset” experiment in Coudreuse & Nurse [7]. See text for explanation. **B.** Time course of MPF activity during the interval 170 < *t* < 360. Note that, after release from G1 reset, MPF activity rises sharply through the thresholds for S and M. The time lag from onset of S phase to onset of M phase is only 16 min, which we interpret as a mitotic catastrophe. All strains carry deletions of the endogenous copies of *cdc2*, *cdc13*, *cig1*, *cig2* and *puc1*.

To simulate a “G1-reset” experiment with the minimal Cdk network model, we multiply MPF activity by a factor of 1/(1+NmPP1), where NmPP1 is the concentration of inhibitor in units of its IC_50_ (concentration that inhibits 50% of the kinase activity). In Fig. 3, we allow a simulated cell to enter S phase at NmPP1 = 0, then set NmPP1 = 1 for 100 min to block the cell in G2 and allow it to accumulate an excess of fusion protein, then set NmPP1 = 20 for 70 min to reset the cell to G1, and finally set NmPP1 = 0 to activate the pool of fusion protein. As can be seen in panel B of the figure, MPF activity increases abruptly after release, triggering a new round of DNA replication at *t* = 320 min. At *t* ≈ 325 min, Wee1 tries unsuccessfully to inhibit the fusion protein, and at *t* = 336 min MPF activity surpasses *θ*_M_, triggering premature entry into mitosis, almost exactly as observed by Coudreuse & Nurse [7]. In this context, conditional mitotic catastrophe happens at twice the normal division size (*m* ≈ 2, Fig. 3). Since the control system is far away from the SNIC bifurcation point (*m* ≈ 0.7), the vertical rise in MPF activity is abrupt and it crosses the thresholds for S and M phases within less than 20 min (Fig. 3). This simulation, modifying the normal dynamic changes in Cdk activity, demonstrates that cells governed by the minimal Cdk network are fundamentally capable of entering mitotic catastrophe, suggesting that specific properties of the Cdk control network in *MCN Δwee1 Δmik1* cells allow for their viable progression through the cell cycle.

### Influence of additional Cdc2:cyclin complexes on *MCN* dynamics

The obvious difference between wild type and *MCN* cells is that G1 and S cyclins (Cig1, Cig2 and Puc1, collectively called CCP) are absent in the *MCN* strain. In fact, even when present, these cyclins are less likely to form complexes with Cdc2, as demonstrated by Coudreuse & Nurse (see Table 1: in the *cdc13-L-cdc2 Δcdc13 Δcdc2* background, there are no significant differences between *CCP^+^* and *ΔCCP* cells).

**Figure 4:**
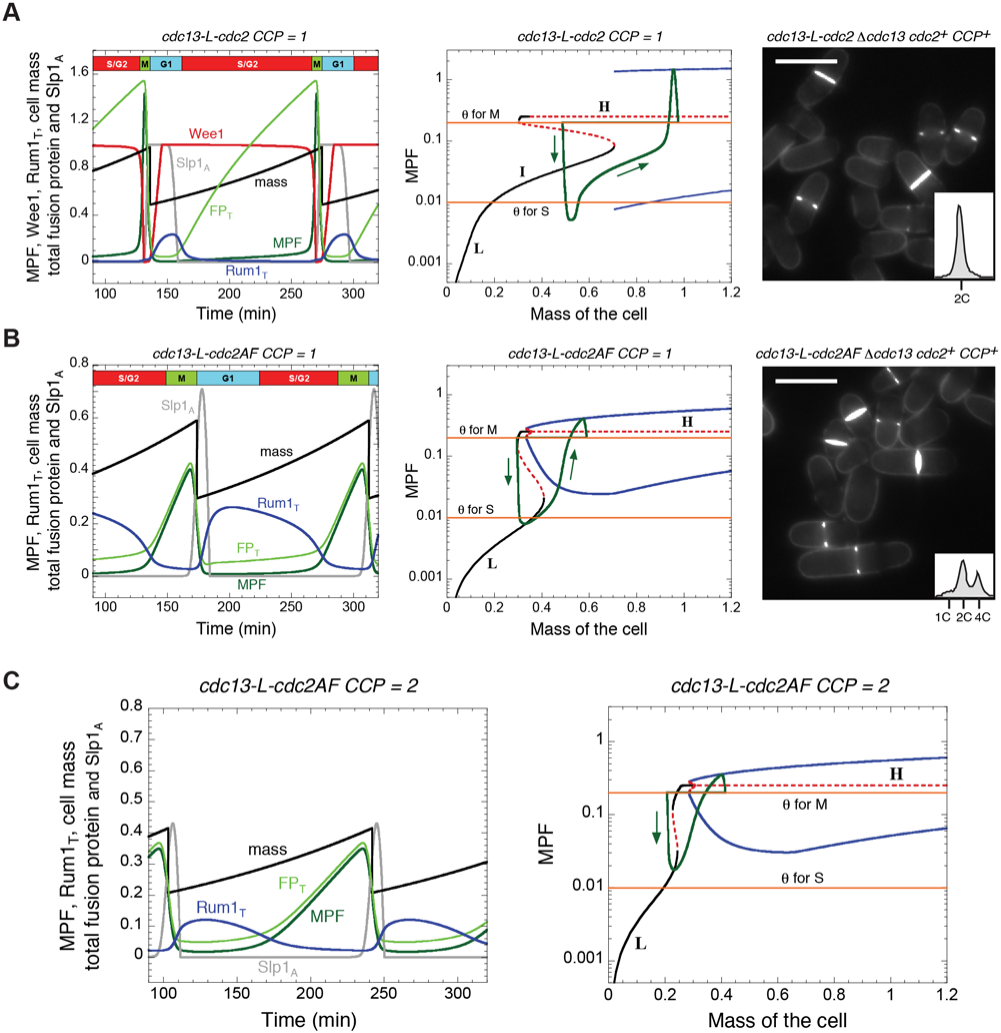
Effects of Cdc2:CCP heterodimer complexes on the dynamics of fission yeast strains carrying the Cdc13-L-Cdc2 fusion protein. The model in S2 Table is modified by adding the following terms for the degradation of Rum1 by CCP-dependent Cdc2 activity: *k_DX_∙CCP∙MPFRum1* to Eq. (5) and *k_DX_∙CCP∙Rum1* to Eq. (6). Those terms are found in Eqs. (1) and (7) as well. Parameter values are as in S3 Table with *k*_DX_ = 1. **A.** Strain *cdc13-L-cdc2 Δcdc13 cdc2^+^ CCP^+^* (CCP = 1). **B.** Strain *cdc13-L-cdc2AF Δcdc13 cdc2^+^ CCP^+^* (CCP = 1). **C.** Strain *cdc13-L-cdc2AF Δcdc13 cdc2AF CCP^+^* (CCP = 2). In the left panels, we plot the time evolution of total fusion protein (light green, FP_T_), active MPF (dark green), active Wee1 (red), total Rum1 (blue), active APC:Slp1 (grey), and cell mass (black). The corresponding bifurcation diagrams for MPF as a function of cell mass are plotted in the middle panels (A and B) and in the right panel (C), with the same conventions as in Fig. 2. While Cdc2:CCP complexes have no effect on the *cdc13-L-cdc2* strain (compare panels in A with Fig. 2A), they have a dramatic effect on the “non-phosphorylable” *cdc13-L-cdc2AF* strain, causing these cells to divide at ~half the size of the *cdc13-L-cdc2* strain. (Panels in Fig. 4B may also be compared with panels in Fig. 2C, because the *MCN-AF* and *MCN Δwee1 Δmik1* strains are nearly identical, as seen in Table 2 Rows 3 and 4.) Right panels in A and B illustrate the blankophor staining of exponentially growing cells in the corresponding conditions together with their DNA content analysis. Scale bar = 10 μm. The strain *cdc13-L-cdc2AF Δcdc13 cdc2^+^ CCP^+^* (CCP = 1) is spontaneously diploidizing, so it is a mix of haploid and diploid cells. However, based on the characterisation of cell width, a substantial fraction of the population appears to be haploid. Therefore, the limited 1C peak supports the shorter G1 in these conditions.

CCP-dependent Cdc2 activity may therefore account for the major differences between wild type and *MCN* cells in the absence of Cdc2 inhibitory phosphorylation. To test this hypothesis, we supplemented our model of *MCN* cells with a “generic” CCP-dependent Cdc2 activity, reflecting the situation in *cdc13-L-cdc2 Δcdc13 cdc2^+^ CCP* ^+^ cells. These additional cyclins account for additional sources of Cdc2 activity throughout the cell cycle, but their temporal patterns are unknown, except for Cig2 [20]. Therefore, in the extended model, this generic Cdc2 activity is represented simply by a parameter, CCP = 1, and its only effect is to promote the phosphorylation and degradation of Rum1 [5, 21]. As a result, the peak of Rum1 accumulation is lowered (Fig. 4A, left panel) and the range of the stable L steady state is restricted (Fig. 4A, middle panel) compared to *MCN* cells (Fig. 2A). However, the cell cycle properties of these two strains are nearly identical in the model (Table 2 Rows 1 and 5), as the cell cycle trajectory remains far from the L steady state. We have confirmed these predictions by examining the phenotype of the corresponding *cdc13-L-cdc2 Δcdc13 cdc2^+^ CCP* ^+^ strain (Fig. 4A right panel and Table 1 Row 10). The only discrepancy is that the observed cell size at division is slightly smaller than *MCN* cells, which could be caused by a small effect of CCPs on mitotic control.

On the other hand, the effect of CCP-dependent Cdc2 activity on cells operating with a non-phosphorylable fusion protein is quite different, as seen in the *cdc13-L-cdc2AF Δcdc13 cdc2^+^ CCP^+^* strain (Fig. 4B). We purposefully consider non-phosphorylable fusion protein rather than *Δwee1 Δmik1* so that only the fusion protein bypasses inhibitory phosphorylation. The simulation clearly suggests the emergence of a “wee” phenotype with reduced cell size at division and extended G1 phase. The I stable steady state disappears from the bifurcation diagram (Fig. 4B, middle panel), similar to the situation in *MCN Δwee1 Δmik1* cells (Fig. 2C, middle panel). The mutant cells (*cdc13-L-cdc2AF Δcdc13 cdc2^+^ CCP^+^*) cycle between a stable L and an unstable H state, replicating their DNA near the L steady state. Notably, although extended, the duration of G1 phase is shorter than in Fig. 2C, as the generic Cdc2:CCP background activity helps the fusion protein to eliminate Rum1 (see also Table 2 Row 6). Consequently, cell size at the SNIC bifurcation in Fig. 4B, middle panel, is about half the value of the SNIC bifurcation point in Fig. 2C, middle panel, so these mutant cells divide at a smaller size than *MCN* cells deleted for *wee1* and *mik1*. These cells (CCP = 1) avoid unconditional mitotic catastrophe because their chromosomes get fully replicated during the 63 min interval when MPF rises from *θ*_S_ to *θ*_M_ (see Table 2). Consistent with these predictions, we find that *cdc13-L-cdc2AF Δcdc13 cdc2^+^ CCP^+^* cells are viable and small with a slightly extended G1 (Fig. 4B, right panel and Table 1 Row 11).

As previously mentioned, replacing *cdc2^+^* by *cdc2AF* in a wild type genetic background causes mitotic catastrophe (Table 1). In the context of our model, we identify such *cdc2AF* cells (in a wild type genetic background) with the strain *cdc13-L-cdc2AF Δcdc13 cdc2AF CCP^+^* (Table 2 Row 8). In contrast to the situation in *cdc13-L-cdc2AF Δcdc13 cdc2^+^ CCP^+^* strain (CCP = 1), Cdc2AF:CCP complexes in these *MCN*-derived cells cannot be inhibited by Wee1 and Mik1 [22]; hence, we set CCP = 2 to model these cells. In the first case (CCP = 1), cells are small and viable (Fig. 4B), whereas in the second case (CCP = 2), cells are small and inviable (Fig. 4C) as they cannot properly re-license the DNA replication origins after cell division (green curve above *θ*_S_ in right panel of Fig. 4C). In addition, in the absence of Wee1- and Mik1-dependent phosphorylation of Cdc2, the S phase checkpoint cannot delay MPF activation, and these cells enter into unconditional mitotic catastrophe at the next mitosis.

**Figure 5:**
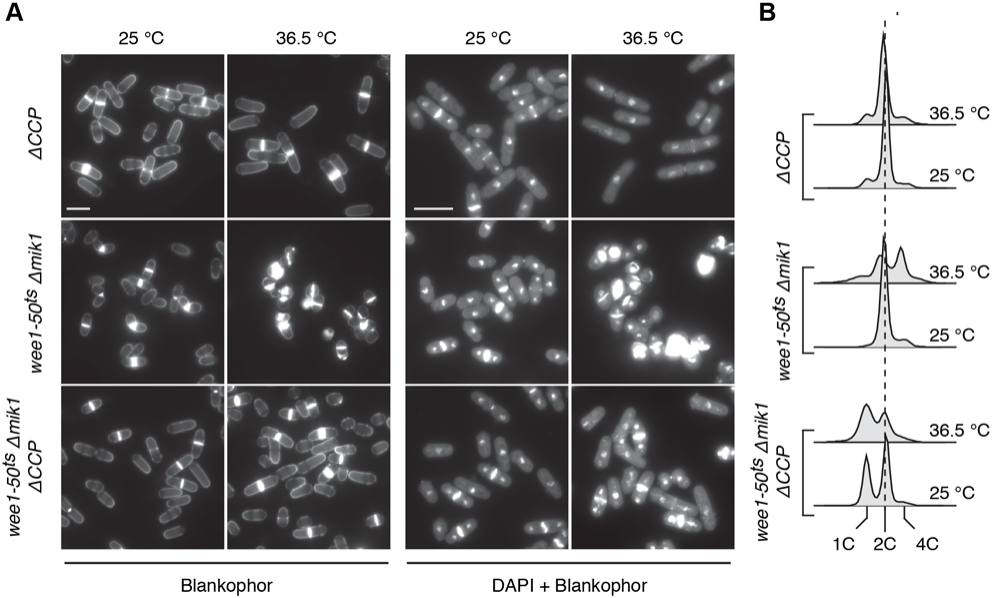
Loss of G1/S cyclins significantly rescues the lethality caused by loss of Cdc2 inhibitory phosphorylation in a wild type background. **A.** Blankophor and DAPI staining of strains at both permissive (25°C) and restrictive (5h at 36.5°C) temperatures for the *wee1–50^ts^* mutation. **B.** DNA content analysis of strains as in A. Note that *wee1–50^ts^ Δmik1* cells at the restrictive temperature tend to diploidize or cut, resulting in an increase in DNA content.

Our analysis of the *MCN* network in the absence of Cdk1 inhibitory phosphorylation therefore suggests that mitotic catastrophe in *cdc2AF* cells in an otherwise wild type background results from unregulated Cdc2 activity mediated by both Cdc13 and CCP cyclins. To test this prediction, we compared a *wee1–50^ts^ Δmik1* strain, which is not viable at the restrictive temperature of 36.5°C, with a *wee1–50^ts^ Δmik1 ΔCCP* strain (Fig. 5). At permissive temperature, both populations of cells were slightly shorter at division than *ΔCCP* cells; we also observed a significantly longer G1 in *wee1–50^ts^ Δmik1 ΔCCP* cells. Strikingly, the absence of G1/S cyclins significantly rescued the effects of loss of Wee1 and Mik1 at restrictive temperature (5h), with a lower incidence of “cut” cells. These results support the conclusions from the model, suggesting that the viability of *MCN-AF* and *MCN Δwee1 Δmik1* cells is not due to an intrinsically lower activity of the fusion system even in the absence of Cdc2 inhibitory phosphorylation. Importantly, they provide compelling evidence that similar to the situation in *MCN* cells, mitotic catastrophe in a wild type background results from the combination of non-regulated Cdc2 activity in association with Cdc13 and the G1/S cyclins.

### Robustness of *MCN* cell cycles in the context of molecular noise

*MCN* cells, *MCN-AF* cells, and *MCN Δwee1 Δmik1* cells exhibit the same mean cell size at division (see Tables 1 and 2), but the size distribution is considerably broader in the absence of inhibitory phosphorylation of Cdc2 [7]. To explore this property, we created a stochastic version of the model and studied the effects of Cdc2 phosphorylation on the robustness of minimal Cdk oscillations towards molecular noise.

We introduced both intrinsic and parametric noise into the model. Intrinsic noise, i.e. molecular fluctuations due to random reaction events, was implemented by using Gillespie’s stochastic simulation algorithm [23]. The reaction steps and propensities of the stochastic model are listed in S4 Table. The reaction rate constants used in the stochastic model are identical to the values used in the deterministic model (see S3 Table). Molecular concentrations in the deterministic model were converted into numbers of molecules by multiplying concentrations by system size (*Ω* = 1000), giving protein numbers in the 100–1000 molecule range. Parametric noise was attributed to variations in total protein levels from one cell to another, due perhaps to differences in the associated rates of transcription [24, 25]. Cell size at division is influenced most sensitively by the expression of the fusion protein and of Rum1 (simulations not shown), so we only considered parametric noise for these proteins.

**Figure 6:**
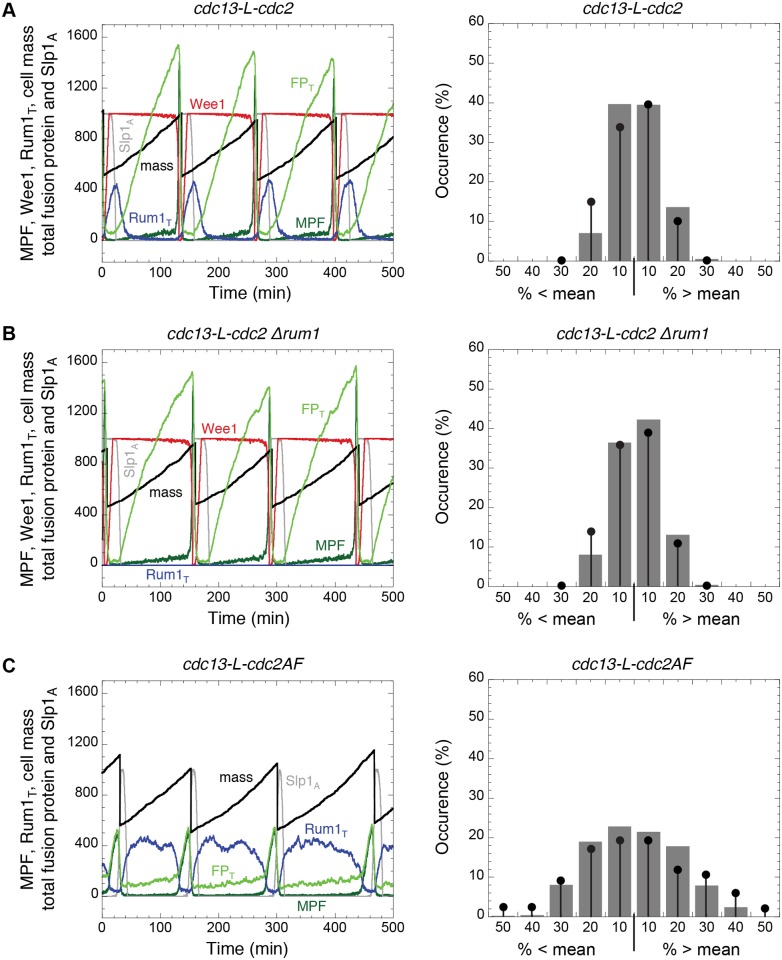
Robustness of MPF oscillations towards molecular noise in *MCN*-derived cells. The stochastic model includes both intrinsic noise (equations in S4 Table) and parametric noise (see Stochastic simulations section). These calculations are done for Ω = 1000, *σ*_SMPF_ = 0.1 and *σ*_SRUM1_ = 0.25; other parameter values as in Fig. 2. **A.** *MCN* strain. **B.** *MCN Δrum1* strain. **C.** *MCN-AF* strain (equivalent to *MCN Δwee1 Δmik1* strain). In the left panels, we plot, as in previous figures, the time evolution of selected proteins over successive cell cycles, to illustrate the magnitude of stochastic fluctuations observed in our model. In the right panels, we plot histograms of cell size at division in the same format as Fig. 5 in [7]. Black circles with bars correspond to the experimental measurements in [7], while the histograms are the proportions calculated with the mathematical model. The number of cells in each of our samples is 500. All strains carry deletions of the endogenous copies of *cdc2*, *cdc13*, *cig1*, *cig2* and *puc1*.

Using both intrinsic and parametric noise, we illustrate the expected variability in cell cycle progression in *MCN*, *MCN Δrum1*, and *MCN-AF* cells in Fig. 6. *MCN Δwee1 Δmik1* cells behave similarly to *MCN-AF* cells, as expected (simulations not shown). Note that if we consider smaller number of molecules, i.e. Ω = 500 or Ω = 200, the cells are still viable. However, the cell size distribution is enlarged in each case and does not correspond to experimental observations from Coudreuse and Nurse [7]. Previous experimental and theoretical studies of cell cycle control have stressed the role of positive feedback loops in promoting robust oscillations in Cdk:cyclin activity [26–31]. Hence, it is no surprise that eliminating Cdc2 phosphorylation from the *MCN* strain, which eliminates the positive feedback loops at the G2/M transition, results in lower amplitude MPF oscillations and greater sensitivity to molecular noise (compare Fig. 6A and B to Fig. 6C). The similarities between these results and the experimental data (Fig. 6 right panels), showing increased variability in cell size at division in the *MCN-AF* cells [7], suggest that molecular noise is an integral part of the cell cycle regulatory circuit.

**Figure 7:**
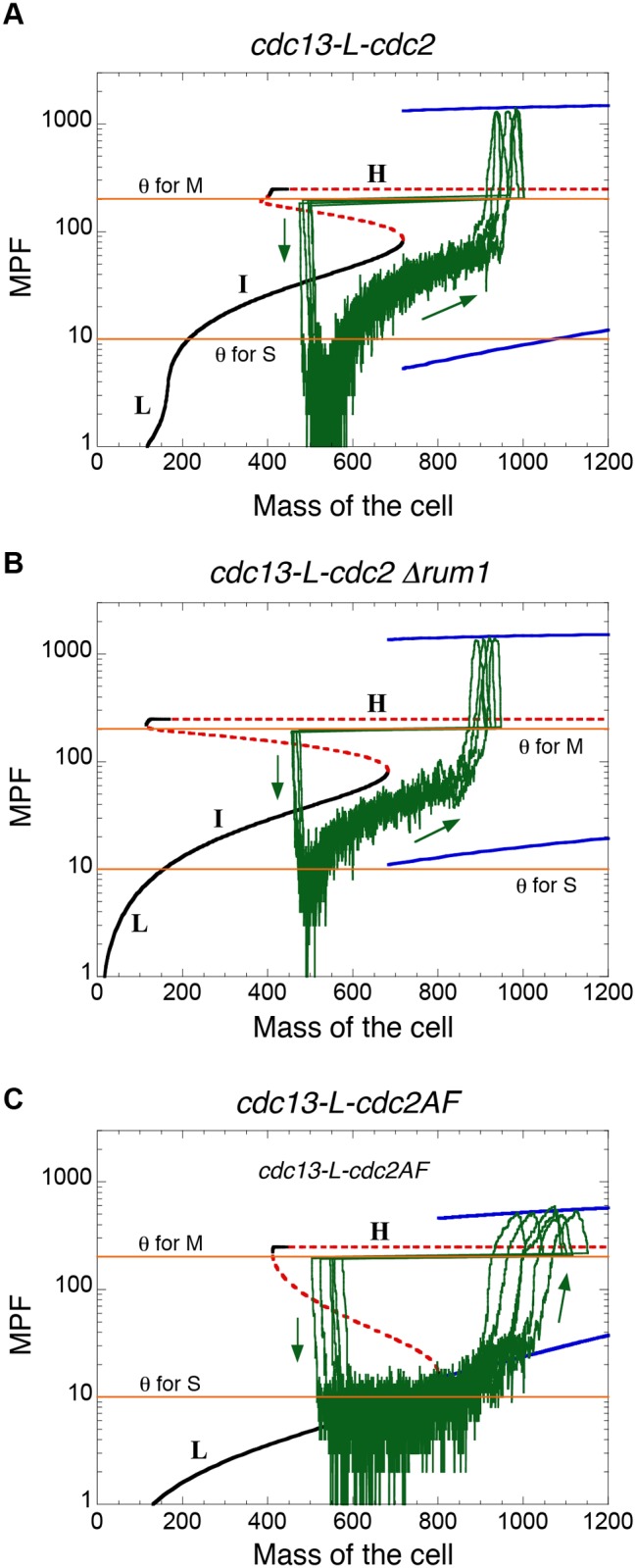
Bifurcation diagrams and stochastic cell cycle trajectories. MPF activity as a function of cell mass for the *MCN* strain (*cdc13-L-cdc2 Δcdc13 Δcdc2 ΔCCP*) in **A**, for the *MCN Δrum1* strain in **B**, and for the *MCN-AF* strain in **C.** Black curves represent stable steady states, red dashed curves define unstable states, and blue curves indicate the envelope (i.e. maxima and minima) of the sustained oscillations. Horizontal lines (orange) are the assumed thresholds of MPF activity needed to promote S and M phases. Superimposed on these bifurcation diagrams are the stochastic cell cycle trajectories of the full Cdk network (green curves). The corresponding stochastic and deterministic simulations are provided in Fig. 6A-C and Fig. 2A-C, respectively. Parameter values for the simulations are as in Fig. 2 for the bifurcation diagrams and Fig. 6 for the stochastic cell cycle trajectories. All strains carry deletions of the endogenous copies of *cdc2*, *cdc13*, *cig1*, *cig2* and *puc1*.

**Figure 8:**
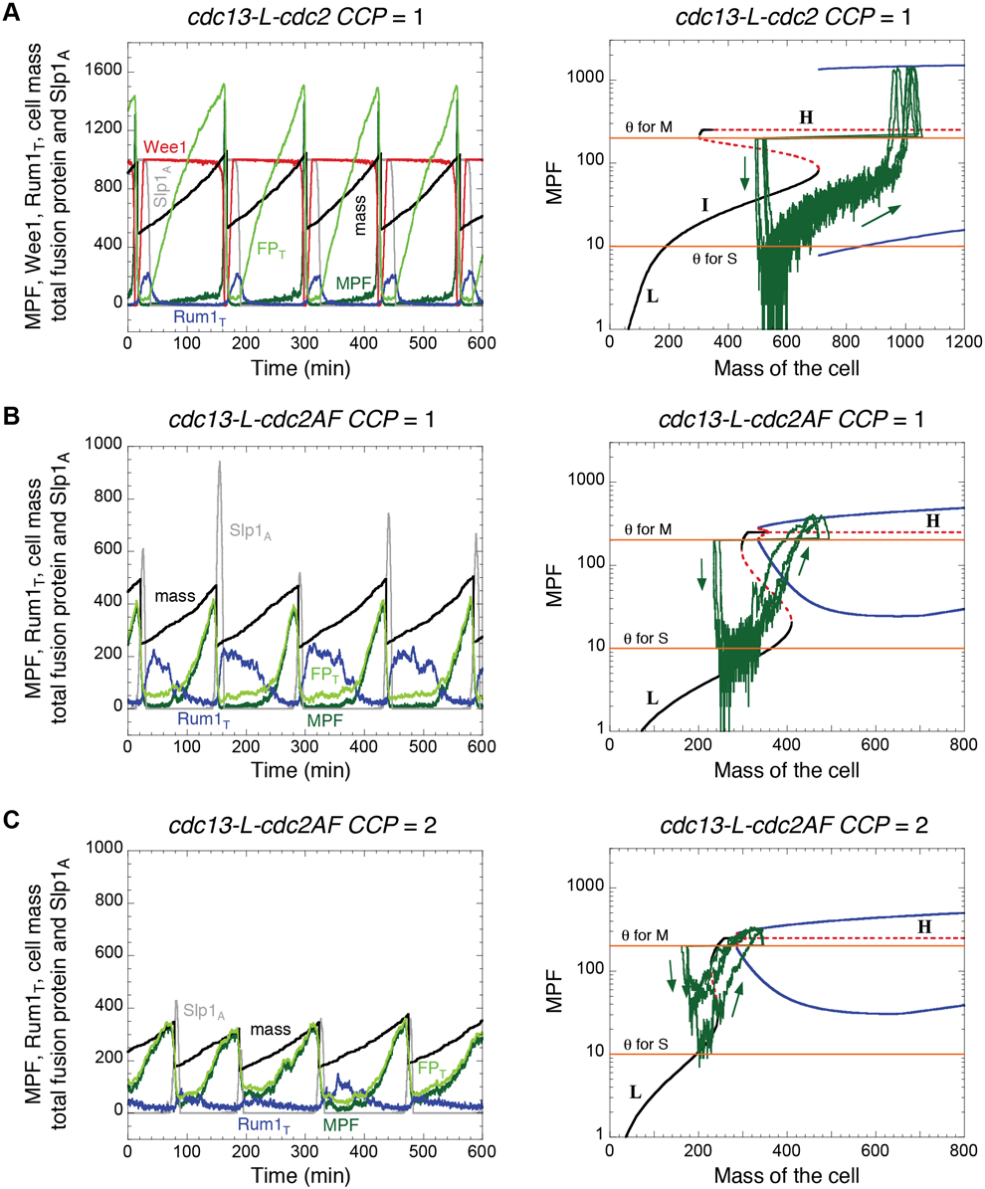
Effects of Cdc2:CCP heterodimeric complexes on the stochastic dynamics of fission yeast strains carrying the Cdc13-L-Cdc2 fusion protein. **A.** Strain *cdc13-L-cdc2 Δcdc13 cdc2^+^ CCP^+^* (CCP = 1). **B.** Strain *cdc13-L-cdc2AF Δcdc13 cdc2^+^ CCP^+^* (CCP = 1). **C.** Strain *cdc13-L-cdc2AF Δcdc13 cdc2AF CCP^+^* (CCP = 2). In the left panels, we plot the stochastic time evolution of total fusion protein (light green, FP_T_), active MPF (dark green), active Wee1 (red), total Rum1 (blue), active APC:Slp1 (grey), and cell mass (black). The bifurcation diagrams for MPF as a function of cell mass together with the stochastic cell cycle trajectories (green curves) are plotted in the right panels. Parameter values for the simulations are as in Fig. 4 for the bifurcation diagrams and Fig. 6 for the stochastic cell cycle trajectories.

With this framework for modelling intrinsic and parametric noise in the minimal Cdk network, we then explored the robustness of our deterministic models of *MCN*-derived cells. In Figs. 7 and 8 we have repeated the deterministic simulations in Figs. 2 and 4, respectively, in the stochastic setting. For five of the six genetic backgrounds, the deterministic model predicts that cells are viable: at cell division, MPF activity drops below *θ*_S_ long enough for a newborn cell to re-license its replication origins, then rises above *θ*_S_ to initiate DNA replication, then (after a sufficiently long time period to complete DNA replication) MPF rises above *θ*_M_ to initiate mitosis, and finally drops below *θ*_M_ to initiate mitotic exit and cell division. In each of these five cases, the stochastic simulations show the same global course of events (Figs. 7 and 8). Therefore, although stochastic fluctuations are expected to introduce considerable variability in cell cycle progression, the minimal Cdk network is sufficiently robust to maintain viability in each of these five mutant strains.

In the sixth case, *cdc13-L-cdc2AF CCP* = 2 (Fig. 4C), the deterministic model predicts that cells do not re-license their replication origins after cell division and die. The stochastic simulations of these cells show that they in fact encounter multiple problems (Fig. 8C). In some cases, MPF activity does not drop low enough for origin re-licensing, a major source of mitotic catastrophe. But, whether or not origins are re-licensed, MPF activity rises so rapidly that these cells are likely to show a cut phenotype, as suggested by the “G1 reset” experiments in [7]. This supports our previous conclusions on the role of unregulated CCP-dependent MPF activity in promoting unconditional mitotic catastrophe and suggests that this phenomenon can be the result of different cellular events (i.e. lack of origin relicensing or fast rise in Cdk activity).

## Discussion

Despite the apparent complexity of cell cycle regulation in eukaryotic cells, a minimal Cdk control network, consisting of an autonomous monomolecular cyclin-Cdk fusion protein, is sufficient to drive normal progression through the entire fission yeast cell cycle [7]. Here, we propose a computational model for this control network (Fig. 1) based on the Cdc13-L-Cdc2 fusion protein (referred to as MPF) and on the notion of “quantitative” control of DNA synthesis and mitosis: MPF initiates DNA replication when its activity exceeds a low threshold (*θ*_S_), and the same MPF activity initiates mitosis when it exceeds a high threshold (*θ*_M_ > *θ*_S_). Our model conforms to previous experimental studies supporting the idea that quantitative–rather than qualitative–changes in Cdk:cyclin heterodimer activity orchestrate the sequence of cell cycle events [1, 4, 6, 7, 32].

Model simulations recapitulate all published phenotypes of mutant fission yeast strains based on the *MCN* genetic background (*cdc13-L-cdc2 Δcdc13 Δcdc2 ΔCCP*). In particular, our model accounts for the unexpected phenotypes of *MCN* cells lacking Cdc2-inhibitory phosphorylation (i.e, *MCN* cells deleted for *wee1* and *mik1*, or *MCN* cells carrying the *cdc13-L-cdc2AF* fusion cassette).

To explore the roles of Cdc2 activity associated with alternative cyclins (Cig1, Cig2 and Puc1; collectively referred to as CCP) on the timing of mitosis, we modified the model slightly and compared the model’s predictions with a set of new minimal strains we generated. Cdc2:CCP complexes were assumed to contribute a background Cdc2 kinase activity in the model equations, specifically targeting Rum1 for degradation. Without this background activity, *MCN-AF* cells (*cdc13-L-cdc2AF Δcdc13 Δcdc2 ΔCCP*) divide at wild type size, but with this background activity *cdc13-L-cdc2AF Δcdc13 cdc2^+^ CCP^+^* cells are “wee” (i.e. divide smaller than wild type cells). This CCP-dependent Cdc2 activity may therefore be at least partly responsible for the smaller size at division of cells with reduced Cdc2 inhibitory phosphorylation in an otherwise wild type background. This led us to propose a new explanation for the mitotic catastrophe that characterizes wild type cells entirely devoid of Cdc2 inhibitory phosphorylation. Our results–that the differences between *cdc2AF* cells and *MCN-AF* cells are consequences of unregulated CCP-dependent Cdc2 activity–suggest that the mitotic catastrophe observed in *cdc2AF* cells may come from excessive accumulation of Cdc2AF:CCP complexes in addition to Cdc2AF:Cdc13, a hypothesis that we have experimentally validated (Fig. 5). In the *MCN* background, Cdc13-L-Cdc2AF is not sufficient to induce mitotic catastrophe, because the lack of inhibitory phosphorylation is counteracted by an elevated Rum1-dependent inhibition of the fusion protein. Our data suggest that this is also the case in a wild type background, and therefore, the Cdc2AF:Cdc13 complex represents only a necessary but not sufficient condition for mitotic catastrophe; entry into mitosis before completion of S phase also involves lack of inhibitory phosphorylation on Cdc2:CCP complexes, which down-regulate the levels of Rum1. This creates a catastrophic situation because replication licensing becomes compromised while entry into mitosis is advanced.

**Figure 9:**
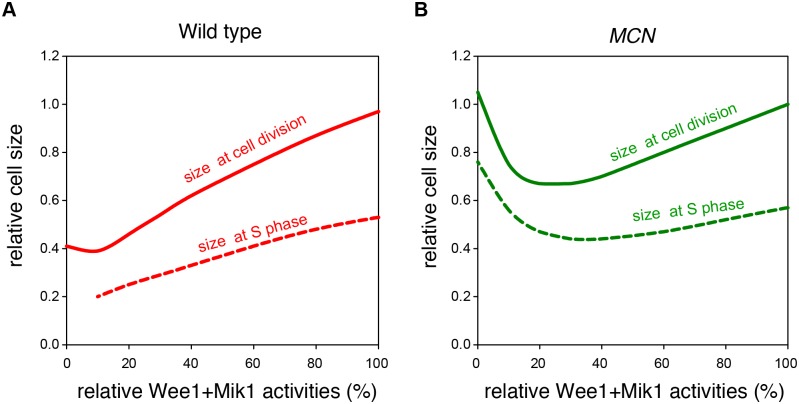
Influence of Wee1+Mik1 activity on cell size in wild type (A) and *MCN* (B) backgrounds. Cell size at division (solid curve) and at S phase (dashed curve) were calculated at different Wee1+Mik1 activity with (A) and without (B) CCP activities. The *MCN* strain carries deletions of the endogenous copies of *cdc2*, *cdc13*, *cig1*, *cig2* and *puc1*.

Our model provides a mechanistic explanation for different physiological consequences of lack of inhibitory Cdc2 phosphorylation in wild type and *MCN* backgrounds (Fig. 9). When the Cdc2 inhibitory phosphorylation network is intact (Wee1+Mik1 = 100%), it determines the critical size for the G2/M transition in both genetic backgrounds, and the S phase size control is cryptic [17]. Subsequently, reduction in Wee1+Mik1 activity leads to a decrease in cell size at division and in cell size at the G1/S transition. In a wild type background (with CCP activities), decreasing Wee1+Mik1 activity causes a decline in cell size at division down to 40% of normal. However, for Wee1+Mik1 < 10%, Cdc2 activity does not drop low enough for efficient licensing of DNA replication origins, and therefore cells divide with catastrophic consequences. In contrast, in *MCN* cells, as Wee1+Mik1 is reduced, the size at division falls to a minimum of 67% of normal, which is reached at Wee1+Mik1 = 20–30%. Further reduction of Wee1+Mik1 activity leads to an increase in the critical size at the G1/S transition caused by persistence of Rum1 and efficient Rum1-dependent inhibition of the unphosphorylated fusion protein, accompanied by a corresponding increase in cell size at division. Therefore, lack of CCP-dependent activities in the *MCN* background provides cells with the opportunity to switch from the traditional size control at G2/M to an S phase size control mechanism, thereby avoiding mitotic catastrophe.

Stochastic simulations of the model show that *MCN-AF* and *MCN Δwee1 Δmik1* cells are more sensitive to molecular noise than cells of the parental *MCN* strain (compare Fig. 6C with Fig. 6A). These results support the idea that positive feedback loops at the G2/M transition are critical to generate robust oscillations of MPF. When the positive feedback loops are abrogated (by the *cdc2AF* allele or by deletion of the inhibitory kinases), populations of *MCN* cells show a broader distribution of cell size at division both experimentally and in simulations. In the presence of both Cdc13- and CCP-dependent Cdc2AF activities, our model suggests that molecular noise induces a range of phenotypes that are consistent with the lethality of Cdc2AF in a wild type background, from cells that do not re-license their replication origins to mitotic catastrophe resulting from too fast rise in Cdk activity (Fig. 8C).

The model proposed here is based on quantitative regulation of the cell cycle, where increasing activity of a single Cdk:cyclin complex drives orderly progression through the successive phases of the DNA replication-division cycle. Our computational view of the quantitative model is supported by experimental evidence from mutant fission yeast cells that rely on a single cyclin-Cdk fusion protein to drive the cell cycle. This situation contrasts with wild type yeast cells (and cells in higher eukaryotes), where different Cdk activities are thought to drive specific cell cycle events. Such “qualitative” regulation of the cell cycle has been modeled in earlier publications [31, 33–41]. Although quantitative regulation of the cell cycle may appear to be an unrepresentative property of an unnatural strain of yeast cells, the absence of phenotype in the *MCN* strain suggests that primeval eukaryotic cells may have controlled their cycle of DNA replication and mitosis using similarly simple mechanisms based on a single protein kinase activity. Later in the evolution of eukaryotes, additional components of the control network may have been introduced to improve its fitness, for instance by increasing robustness through redundancy. Nonetheless, even in higher eukaryotes, simplified versions of the cell cycle control system, with some of this redundancy removed, can still function reliably [42, 43].

## Materials and Methods

### Mathematical modelling

To develop a mathematical model of the minimal Cdk network, we assume that the fusion protein is regulated similarly to the Cdc2:Cdc13 heterodimeric complex in wild type cells, which we have previously modeled [44–47]. That is, we assume that the activity of Cdc13-L-Cdc2 is controlled by 1) inhibitory phosphorylation of the Cdc2 moiety, 2) proteolytic degradation of the fusion protein mediated by the cyclin destruction box, and 3) binding of the stoichiometric Cdk inhibitor Rum1 (Fig. 1). For simplicity, although the Cdc13-L-Cdc2 fusion protein and the normal Cdc2:Cdc13 heterodimeric complex have SPF as well as MPF activities, we shall refer to both as MPF (M-phase Promoting Factor), because only Cdc13-dependent Cdc2 activity can bring about M phase in fission yeast [4].

Inhibitory phosphorylation of MPF by Wee1 and Mik1 (on T14 and Y15 of Cdc2) results in the accumulation of a less active form of MPF, labeled here as MPF_P_ (Fig. 1, S/G2 module). We assume that the activity of MPF_P_ is 5% of MPF activity [48, 49]. For our study, the distinction between Wee1 and Mik1 is not necessary, and we therefore refer to them together as Wee1 (hence, *Δwee1* in our simulations is equivalent to *Δwee1 Δmik1* in the experiments). MPF inhibitory phosphorylations are antagonized by a protein phosphatase, Cdc25, that dephosphorylates T14P and Y15P of Cdc2 and thereby releases MPF activity [12]. Importantly, feedback loops are built into the system, as the activities of Wee1 and Cdc25 are themselves regulated by MPF (and to a lesser extent by MPF_P_). As indicated in Fig. 1 (S/G2 module), Wee1 is phosphorylated and inactivated by MPF, whereas Cdc25 is phosphorylated and activated by MPF, thus establishing a double-negative feedback loop (Wee1⊣ MPF⊣ Wee1) and a positive feedback loop (Cdc25 → MPF → Cdc25). These two feedback loops create a bistable switch that is responsible for abrupt MPF activation at the G2/M transition [50].

At the end of M phase, Cdc13 is ubiquitinated by the Anaphase Promoting Complex (APC, also known as Cyclosome), which tags the fusion protein of the minimal module for rapid proteasomal degradation (Fig. 1, M module). Initial degradation of the fusion protein is mediated by the APC in conjunction with Slp1 (the fission yeast orthologue of Cdc20) [51]. This system introduces a negative feedback loop, as MPF promotes its own degradation by activating APC:Slp1, a feature that is common to all eukaryotes. Both theoretical arguments [52] and experimental evidence [53, 54] suggest that this negative feedback loop is time-delayed, but the underlying molecular mechanism of this delay is unclear. For this study, we generate a time delay by inserting an intermediary enzyme (IE) between MPF and APC:Slp1, as in earlier models [50] (see Eq. (4) in S2 Table).

We use Goldbeter-Koshland kinetics [55] to describe the ultrasensitive activation and inactivation of enzymes controlling the phosphorylation of MPF (Wee1 and Cdc25) and the degradation of Cdc13 (IE and APC). (See equations (3), (4), (8) and (9) in S2 Table.)

In G1 phase, MPF activity is kept low by a stoichiometric Cdk-inhibitor, Rum1, which binds to Cdc13-L-Cdc2 and blocks the catalytic activity of the fusion protein (Fig. 1, G1 module). MPF activity is also kept low by APC-dependent degradation of the fusion protein, in conjunction with Ste9 (fission yeast orthologue of Cdh1, also known as Srw1) [18]. Since Rum1 and Ste9 are both active during G1 phase of the fission yeast cell cycle, we lump them together as G1 Cdk-inhibitory activity (in Fig. 1), attributing G1-specific Cdc13 degradation to Rum1 [21].

Not only does the Cdk-inhibitor Rum1 bind to Cdc13-L-Cdc2 and quench its activity [56], but Rum1 is also phosphorylated by active Cdc2 on multiple sites, targeting it for rapid ubiquitin-dependent degradation [57]. Therefore, Rum1 is both an inhibitor and a substrate of Cdc13-L-Cdc2. Elsewhere, we have argued [16] that this relationship between Rum1 and MPF is characterized by a particular network motif (SIMM = Substrate Inhibitor Multiply Modified) that generates an abrupt G1/S transition in eukaryotic cells. In Fig. 1 (G1 module), we implement the SIMM motif for distributive, two-step phosphorylation of Rum1 by MPF.

In wild type cells, G1 phase is short, presumably because the CCP cyclins (Cig1, Cig2 and Puc1), in combination with Cdc2, effectively phosphorylate Rum1 and Ste9, leading to their rapid degradation and inactivation, respectively. These “starter kinase” activities induce the SIMM motif to undergo an irreversible transition from G1 into S phase. In *MCN* cells, which lack CCP-dependent Cdc2 activity, the role of starter kinase must be played by MPF_P_ (i.e., the tyrosine-phosphorylated form of Cdc2-L-Cdc13). This conclusion suggests that MPF_P_ is not effectively inhibited by Rum1 and that MPF_P_, despite its feeble kinase activity (compared to MPF), is able to phosphorylate Rum1 and mark it for degradation.

To put all these ideas together in a consistent fashion, we have made a number of assumptions in writing the differential equations in S2 Table and choosing the parameter values in S3 Table. In Eqs. (5)–(7) in S2 Table, we have implemented a SIMM motif for the distributive, two-step phosphorylation of Rum1 by MPF. In this scheme, the first phosphorylation of Rum1 by MPF is described by a Michaelis-Menten mechanism with tight binding of Rum1 to the kinase [57, 58] and slow phosphorylation of the enzyme-bound substrate (MPF:Rum1 ➔ MPF + Rum1_P_). In this case, the Michaelis constant of the enzyme (Cdc13-L-Cdc2) is small, *K*_m_ = (*k*_DISS_ + *k*_IRUM1_)/*k*_ASS_ ≈ 0.02 << [Rum1]_total_ ≈ 0.4 (in G1 phase), and the turnover number of the enzyme-substrate complex, *k*_IRUM1_ = 2 min^−1^, is small compared to the dephosphorylation of Rum1_P_, *k*_ARUM1_ = 35 min^−1^. Hence, most of the enzyme (MPF) is tied up in the enzyme-substrate complex. In this context, Rum1 reduces the availability of Cdc2 for phosphorylating other substrates (i.e., Rum1 is a stoichiometric inhibitor of Cdc2).

After the first phosphorylation, Rum1_P_ can be either dephosphorylated by a phosphatase (Rum1_P_ ➔ Rum1) or undergo a second phosphorylation by MPF (Rum1_P_ ➔ Rum1_P2_), followed by rapid degradation (Rum1_P2_ ➔ degradation). According to the SIMM concept, the phosphorylation of Rum1_P_ by MPF has a large Michaelis constant (*K*_m2_ >> [Rum1]_total_) and a large turnover number (*k*_2_ >> *k*_dissociation_). Therefore, the phosphorylation of Rum1_P_ can be described by second-order mass-action kinetics (*k*_DRUM1P_ = 250 CU^−1^ min^−1^, where CU = concentration unit for MPF and Rum1), neglecting the concentration of enzyme-substrate complexes.

Regarding the phosphorylation of Rum1 by MPF_P_, we assume that both the first and the second phosphorylation steps are governed by second-order mass-action kinetics. Even though MPF_P_ is a less active kinase than MPF, it must be more efficient at phosphorylating Rum1, because the phenotype of the *MCN* strain suggests that MPF_P_ is an effective starter kinase.

How can this be? As a possible explanation (in the absence of any experimental evidence one way or another), we suggest that the phosphorylation of Rum1 by Cdc2 kinase in the enzyme-substrate complex is a two-step process. When Rum1 first binds to Cdc2, the kinase is unable to phosphorylate Rum1. The complex must undergo a conformational transition before the kinase can do its job. Hence, the turnover number for Rum1 phosphorylation by MPF can be written *k*_2_ = *f*∙*k*_p_, where *f* is the fraction of Rum1:MPF complexes in the phosphorylable form and *k*_p_ is the probability per unit time that MPF carries out the phosphorylation of Rum1 in this form. According to S3 Table, *k*_2_ ≡ *k*_IRUM1_ = 2 min^−1^. The Michaelis constant for this reaction is *K*_m_ ≈ *f*∙*k*_p_/*k*_ASS_ = 0.02 CU, because *k*_DISS_ is very small (according to S3 Table). For MPF_P_-catalyzed phosphorylation of Rum1, the turnover number is *k*_2_^’^ = *f* ^’^∙*k*_p_^’^ ≈ *α*∙*k*_p_, where *α* = 0.05 = fractional activity of MPF_P_ compared to MPF and we have assumed that *f* ^’^ ≈ 1 for the Rum1:MPF_P_ complex. The Michaelis constant for the MPF_P_-catalyzed reaction is *K*_m_^’^ ≈ *α*∙*k*_p_/*k*_ASS_^’^, assuming that *k*_DISS_ is also very small for the Rum1:MPF_P_ complex. Assuming that *K*_m_^’^ >> 1 CU, we assure that the first phosphorylation of Rum1 by MPF_P_ follows second-order mass-action kinetics, with rate constant = *k*_2_^’^/*K*_m_^’^ = *k*_ASS_^’^ ≡ *k*_I2RUM1_ = 50 CU^−1^ min^−1^. All these conditions can be satisfied if *f* << *α*/25 = 0.002.

In addition, we need a mechanism for coupling the minimal Cdk network to cell growth. In previous models [44, 46], we have implemented this idea by letting the effective concentration of Cdk:cyclin complexes increase with cell size. The idea behind this assumption is that the number of cyclin molecules increases steadily as cells grow, as is true for most cellular proteins, and that Cdk:cyclin complexes then move into the nucleus where their concentration at local defined sites of action increases in proportion to cell size. Note that changes in the total amount of Cdc13-L-Cdc2 throughout the cell cycle do not simply reflect cell mass increase due to the cell cycle-dependent regulation of Cdc13-L-Cdc2 amount by APC and Rum1. For simplicity, we assume that cell size increases exponentially and is divided in half at cell division (when MPF activity drops below 0.2, in the arbitrary units adopted by the model).

Finally, each phosphorylation reaction in the model is reversed by a dephosphorylation step catalyzed by a phosphatase (Fig. 1), and we assume that these phosphatases have constant activities. This assumption is clearly an oversimplification, because some of these phosphatases are known to be cell cycle regulated [59].

All simulations were performed by means of the software packages XPPAUTO (http://www.math.pitt.edu/~bard/xpp/xpp.html) and MATLAB.

**Stochastic simulations**. At birth, each cell is assigned a value for *k*_SMPF_ and *V*_SRUM1_ from a uniform distribution centered on the deterministic values given in S3 Table. More precisely, *k*_SMPF_ = (1 + *σ*_SMPF_*r)*0.05, where *σ*_SMPF_ is a constant (0 < *σ*_SMPF_ < 1), *r* is a uniform random deviate on [−1, 1], and *k*_SMPF_ = 0.05 is the deterministic value. *V*_SRUM1_ is computed from a similar equation, with *σ*_SRUM1_ determining the cell-to-cell variations in the rate of synthesis of Rum1.

To estimate *σ*_SMPF_ and *σ*_SRUM1_, we performed a series of stochastic simulations for different values of these parameters, in each case calculating the distribution of cell size at division for *MCN* and *MCN-AF* cells. Comparing these simulated distributions to the observed distributions in Fig. 5C of Coudreuse & Nurse [7], we conclude that *σ*_SMPF_ = 0.1 and *σ*_SRUM1_ = 0.25. These simulations suggest that production of Rum1 is considerably more variable from cell to cell than production of the fusion protein.

### Experimental procedures

**Strains and growth conditions**. Standard methods for fission yeast manipulation were used [60, 61]. Strains described in this study are listed in S5 Table. Experiments were carried out in minimal medium plus supplements (EMM4S) at 32°C, except when otherwise indicated. The different Cdk fusion modules and gene deletions are as previously described [7]. The deletions of the G1/S cyclins in Fig. 5 are full deletions of the ORFs of the genes using antibiotic resistance cassettes (see S5 Table).

The synthetic lethality of the MCN-AF cells in combination with deletion of the Cdc2 inhibitor Rum1 was determined through genetic crosses. MCN-AF cells were crossed with Δrum1 cells. Given that either Δcdc2 or Δcdc13 in the absence of the fusion protein results in lethality and that the AF mutation gives rise to a generally higher frequency of non-germinating spores, we could not assess the Mendelian segregation of alleles. Instead, we identified clones deleted for cdc13 (Kanamycin resistance) after tetrad dissection of the cross. These cells must carry the AF fusion protein to be viable, which was verified by PCR. Those strains were then screened by PCR for the presence of the rum1 deletion. None of the 61 clones tested carried both the AF and Δrum1 alterations.

Next, to address the possibility that the MCN-AF Δrum1 strain grows poorly and shows a higher incidence of cell death, random spore analysis of the above cross was performed in the presence of Phloxin B, which is retained in unhealthy and dead cells. Phloxin B-stained small red clones which were deleted for *cdc13* and contained the AF fusion were genotyped by PCR for *Δrum1*. None of the 48 colonies tested were positive for the *rum1* deletion.

Finally, we isolated diploid cells carrying at least one copy of the AF fusion cassette and heterozygous for Δrum1. Following sporulation of this strain, we identified 45 haploid clones containing the cdc13 deletion and AF fusion, and none of them were deleted for rum1.

In total, we have tested 154 haploid strains carrying Δcdc13 and the AF fusion, and none of these contained the rum1 deletion. As we had no difficulty generating a strain with both the normal fusion protein and Δrum1 (which are approximately 0.8 Mb apart), we conclude that the AF fusion cassette and rum1 deletion are synthetically lethal.

**Microscopy**. For cell size measurement and live cell imaging, cells were stained with Blankophor (MP Biohemicals). Images were then acquired with Visiview (Visitron Systems GmbH) using a Nikon Eclipse Ti epifluorescence microscope and a Hamamatsu Orca Flash 4.0 camera. Cell size was determined with ImageJ (National Institutes of Health) using the Pointpicker plug-in. For DAPI + Blankophor staining, cells were heat-fixed on microscope slides, stained with a DAPI/Blankophor solution (1:4) and imaged with Visiview (Visitron Systems GmbH) using a Zeiss Axio Observer microscope equipped with a Hamamatsu Orca Flash 4.0 camera.

**Flow cytometry**. DNA content analysis within a population of cells was performed by flow cytometry using 70% ethanol-fixed and propidium-iodide-stained cells (2mg/ml PI in 50 mM sodium citrate after treatment with RNAse A) and a BD FACSCalibur or Accuri C6 flow cytometer.

## Supporting Information

S1 Table Variables of the model. (DOCX)

S2 Table Differential equations of the model. (DOCX)

S3 Table Parameters of the model. (DOCX)

S4 Table Stochastic version of the model. (DOCX)

S5 Table Strains used in this study. (DOCX)
