# Supplementary material for: Cell Cycle Control by a Minimal Cdk Network
Source: PLoS Comput Biol. 2015 Feb 6;11(2):e1004056. doi: 10.1371/journal.pcbi.1004056 (PMC4319789; doi:10.1371/journal.pcbi.1004056)
Supplement: S1 Table — (DOCX) [file pcbi.1004056.s001.docx]

**Table S1. Variables of the model**

| Symbol | Definition |
| --- | --- |
| MPF | Active Cdk:cyclin complex (fusion protein Cdc13-L-Cdc2) |
| MPF_P_ | Phosphorylated form of the Cdk:cyclin complex, preMPF |
| Slp1_A_ | Active, phosphorylated form of the anaphase-promoting complex, APC:Slp1 |
| IE_A_ | Active form of an intermediate enzyme, which in turn activates APC:Slp1 |
| MPFRum1 | Inactive complex between MPF and Rum1 |
| Rum1 | Cyclin-dependent Kinase Inhibitor, CKI, in fission yeast |
| Rum1_P_ | Inactive, phosphorylated form of Rum1 |
| Wee1 | Active, dephosphorylated form of the kinase Wee1 |
| Cdc25_P_ | Active, phosphorylated form of the phosphatase Cdc25 |
| Mass | Mass of the cell |
