# Supplementary material for: Cell Cycle Control by a Minimal Cdk Network
Source: PLoS Comput Biol. 2015 Feb 6;11(2):e1004056. doi: 10.1371/journal.pcbi.1004056 (PMC4319789; doi:10.1371/journal.pcbi.1004056)
Supplement: S2 Table — (DOCX) [file pcbi.1004056.s002.docx]

**Table S2. Differential equations of the model**

| $\begin{matrix} \frac{dMPF}{dt}=k_{\text{SMPF}}\cdot Mass-k_{\text{WEE1}}\cdot MPF+k_{\text{CDC25}}\cdot MPF_{\text{P}}-\left(k_{\text{D1CYC}}+k_{\text{D2CYC}}\cdot Slp1_{\text{A}}\right)\cdot MPF \\ -k_{\text{ASS}}\cdot Rum1\cdot MPF+\left(k_{\text{DISS}}+k_{\text{DRUM1}}+k_{\text{IRUM1}}\right)\cdot MPFRum1+k_{\text{DX}}\cdot CCP\cdot MPFRum1 \end{matrix}$ | [1] |
| --- | --- |
| $\frac{dMPF_{\text{P}}}{dt}=k_{\text{WEE1}}\cdot MPF-k_{\text{CDC25}}\cdot MPF_{\text{P}}-\left(k_{\text{D1CYC}}+k_{\text{D2CYC}}\cdot Slp1_{\text{A}}\right)\cdot MPF_{\text{P}}$ | [2] |
| $\frac{dSlp1_{\text{A}}}{dt}=k_{\text{1SLP1}}\cdot IE_{\text{A}}\cdot\frac{Slp1}{J_{\text{1SLP1}}+Slp1}-V_{\text{2SLP1}}\cdot\frac{Slp1_{\text{A}}}{J_{\text{2SLP1}}+Slp1_{\text{A}}}$ | [3] |
| $\frac{dIE_{\text{A}}}{dt}=k_{\text{1IE}}\cdot\left(MPF+\alpha\cdot MPF_{\text{P}}\right)\cdot\frac{IE}{J_{\text{1IE}}+IE}-V_{\text{2IE}}\cdot\frac{IE_{\text{A}}}{J_{\text{2IE}}+IE_{\text{A}}}$ | [4] |
| $\begin{matrix} \frac{dMPFRum1}{dt}=k_{\text{ASS}}\cdot Rum1\cdot MPF-\left(k_{\text{DISS}}+k_{\text{DRUM1}}+k_{\text{IRUM1}}+k_{\text{DMPFRUM1}}\right)\cdot MPFRum1 \\ -k_{\text{DX}}\cdot CCP\cdot MPFRum1 \end{matrix}$ | [5] |
| $\begin{matrix} \frac{dRum1}{dt}=V_{\text{SRUM1}}-k_{\text{ASS}}\cdot Rum1\cdot MPF+\left(k_{\text{DISS}}+k_{\text{DMPFRUM1}}\right)\cdot MPFRum1 \\ -k_{\text{I2RUM1}}\cdot MPF_{\text{P}}\cdot Rum1+k_{\text{ARUM1}}\cdot Rum1_{\text{P}}-k_{\text{DRUM1}}\cdot Rum1-k_{\text{DX}}\cdot CCP\cdot Rum1 \end{matrix}$ | [6] |
| $\begin{matrix} \frac{dRum1_{\text{P}}}{dt}=k_{\text{IRUM1}}\cdot MPFRum1+k_{\text{I2RUM1}}\cdot MPF_{\text{P}}\cdot Rum1-k_{\text{ARUM1}}\cdot Rum1_{\text{P}} \\ -k_{\text{DRUM1P}}\cdot\left(MPF+\alpha\cdot MPF_{\text{P}}\right)\cdot Rum1_{\text{P}}-k_{\text{DRUM1}}\cdot Rum1_{\text{P}} \\ +k_{\text{DX}}\cdot CCP\cdot Rum1+k_{\text{DX}}\cdot CCP\cdot MPFRum1-k_{\text{DX}}\cdot CCP\cdot Rum1_{\text{P}} \end{matrix}$ | [7] |
| $\frac{dWee1}{dt}=V_{\text{WEE1}}\cdot\frac{Wee1_{\text{P}}}{J_{\text{1WEE1}}+Wee1_{\text{P}}}-k_{\text{WEE1}}\cdot\left(MPF+\alpha\cdot MPF_{\text{P}}\right)\cdot\frac{Wee1}{J_{\text{2WEE1}}+Wee1}$ | [8] |
| $\frac{dCdc25_{\text{P}}}{dt}=k_{\text{CDC25}}\cdot\left(MPF+\alpha\cdot MPF_{\text{P}}\right)\cdot\frac{Cdc25}{J_{\text{1CDC25}}+Cdc25}-V_{\text{CDC25}}\cdot\frac{Cdc25_{\text{P}}}{J_{\text{2CDC25}}+Cdc25_{\text{P}}}$ | [9] |
| $\frac{dMass}{dt}=\mu\cdot Mass$ | [10] |
| with the following definitions and conservation equations:  $k_{\text{WEE1}}=k_{\text{1WEE1}}\cdot Wee1_{\text{T}}+\left(k_{\text{2WEE1}}-k_{\text{1WEE1}}\right)\cdot Wee1$;  $k_{\text{CDC25}}=k_{\text{1CDC25}}\cdot Cdc25_{\text{T}}+\left(k_{\text{2CDC25}}-k_{\text{1CDC25}}\right)\cdot Cdc25_{\text{P}}$;  $Wee1_{\text{P}}=Wee1_{\text{T}}-Wee1$; $Cdc25=Cdc25_{\text{T}}-Cdc25_{\text{P}}$; $IE=IE_{\text{T}}-IE_{\text{A}}$; $Slp1=Slp1_{\text{T}}-Slp1_{\text{A}}$  | |
