# Supplementary material for: Cell Cycle Control by a Minimal Cdk Network
Source: PLoS Comput Biol. 2015 Feb 6;11(2):e1004056. doi: 10.1371/journal.pcbi.1004056 (PMC4319789; doi:10.1371/journal.pcbi.1004056)
Supplement: S3 Table — (DOCX) [file pcbi.1004056.s003.docx]

**Table S3. Parameters of the model**

| Symbol | Definition | Numerical value^*^ | |
| --- | --- | --- | --- |
| *k*_SMPF_ | Rate of synthesis of Cdk-cyclin fusion protein, MPF | 0.05 | |
| *k*_ASS_ | Bimolecular rate constant for binding of Rum1 to MPF | 100 | |
| *k*_DISS_ | Rate constant for dissociation of complex between Rum1 and MPF | 0.0025 | |
| *k*_DRUM1_ | Rate constant for Rum1 degradation | 0.125 | |
| *k*_IRUM1_ | Rate constant for the phosphorylation of Rum1 by MPF | 2 | |
| *k*_I2RUM1_ | Rate constant for the phosphorylation of Rum1 by MPF_P_ | 50 | |
| *k*_1SLP1_ | Rate constant for the activation, by phosphorylation, of Slp1 | 0.8 | |
| *V*_2SLP1_ | Maximum inactivation rate, by dephosphorylation, of Slp1_A_ | 0.2 | |
| *J*_1SLP1_ | Michaelis constant for Slp1 phosphorylation | 0.001 | |
| *J*_2SLP1_ | Michaelis constant for Slp1_A_ dephosphorylation | 0.001 | |
| *k*_1IE_ | Rate constant for the activation, by phosphorylation, of IE | 0.2 | |
| *V*_2IE_ | Maximum inactivation rate, by dephosphorylation, of IE_A_ | 0.05 | |
| *J*_1IE_ | Michaelis constant for IE phosphorylation | 0.001 | |
| *J*_2IE_ | Michaelis constant for IE_A_ dephosphorylation | 0.001 | |
| *k*_DMPFRUM1_ | Rate constant for the degradation of MPF, which is in the complex MPF:Rum1 | 0.35 | |
| *V*_SRUM1_ | Rate of synthesis of Rum1 | 0.06 | |
| *k*_ARUM1_ | Rate constant for dephosphorylation of Rum1_P_ | 35 | |
| *k*_DRUM1P_ | Rate constant for degradation of Rum1_P_, promoted by MPF | 250 | |
| *V*_WEE1_ | Maximum activation rate, by dephosphorylation, of Wee1_P_ | 0.125 | |
| *k*_WEE1_ | Rate constant for Wee1 phosphorylation by MPF | 0.625 | |
| *J*_1WEE1_ | Michaelis constant for Wee1_P_ dephosphorylation | 0.01 | |
| *J*_2WEE1_ | Michaelis constant for Wee1 phosphorylation | 0.01 | |
| *Wee1*_T_ | Total concentration of the kinase Wee1 | 1 | |
| *k*_CDC25_ | Rate constant for Cdc25 phosphorylation by MPF | 1 | |
| *V*_CDC25_ | Maximum inactivation rate, by dephosphorylation, of Cdc25_P_ | 0.2 | |
| *J*_1CDC25_ | Michaelis constant for Cdc25 phosphorylation | 0.01 | |
| *J*_2CDC25_ | Michaelis constant for Cdc25_P_ dephosphorylation | 0.01 | |
| *μ* | Growth rate of the cell | 0.005 | |
| *k*_1WEE1_ | Rate constant for phosphorylation of MPF by Wee1_P_ | 0.05 | |
| *k*_2WEE1_ | Rate constant for phosphorylation of MPF by Wee1 | 2.5 | |
| *k*_1CDC25_ | Rate constant for dephosphorylation of MPF_P_ by Cdc25 | 0.05 | |
| *k*_2CDC25_ | Rate constant for dephosphorylation of MPF_P_ by Cdc25_P_ | 2.5 | |
| *k*_D1CYC_ | Rate constant for degradation of MPF by Slp1 | 0.0235 | |
| *k*_D2CYC_ | Rate constant for degradation of MPF by Slp1_A_ | 0.75 | |
| *Cdc25*_T_ | Total concentration of the phosphatase Cdc25 | 1 | |
| *IE*_T_ | Total concentration of the intermediate enzyme IE | 1 | |
| *Slp1*_T_ | Total concentration of the protein Slp1 | 1 | |
| α | Partial activity of MPF_P_ to promote the degradation of Rum1_P_ and the phosphorylation of Rum1, Wee1, Cdc25 and IE | 0.05 | |
| **Addition of a background Cdk activity dependent on the G1 cyclins (CCP), which promotes the phosphorylation of Rum1** | | | |
| *k*_DX_ | Rate constant for the phosphorylation of Rum1 by Cdc2:CCP | | 1 |

^*^ Units for the parameters are min^-1^ for rate constants (*k*’s and *V*’s) and dimensionless concentrations for the Michaelis constants (*J*’s) and total concentrations. *µ* has units
min^-1^, and *α* is a dimensionless number.
