# Supplementary material for: Cell Cycle Control by a Minimal Cdk Network
Source: PLoS Comput Biol. 2015 Feb 6;11(2):e1004056. doi: 10.1371/journal.pcbi.1004056 (PMC4319789; doi:10.1371/journal.pcbi.1004056)
Supplement: S4 Table — (DOCX) [file pcbi.1004056.s004.docx]

**Table S4. Stochastic version of the model**

| Reaction number | Reaction | Propensity of reaction |
| --- | --- | --- |
| 1 |  | $w_{1}=k_{\text{SMPF}}\cdot Mass$ |
| 2 |  | $w_{2}=\left[\frac{k_{\text{1WEE1}}}{\Omega}\cdot Wee1_{\text{T}}+\left(\frac{k_{\text{2WEE1}}-k_{\text{1WEE1}}}{\Omega}\right)\cdot Wee1\right]\cdot MPF$ |
| 3 |  | $w_{3}=\left[\frac{k_{\text{1CDC25}}}{\Omega}\cdot Cdc25_{\text{T}}+\left(\frac{k_{\text{2CDC25}}-k_{\text{1CDC25}}}{\Omega}\right)\cdot Cdc25_{\text{P}}\right]\cdot MPF_{\text{P}}$ |
| 4 |  | $w_{4}=\left[\frac{k_{\text{D1CYC}}}{\Omega}\cdot Slp1_{\text{T}}+\left(\frac{k_{\text{D2CYC}}-k_{\text{D1CYC}}}{\Omega}\right)\cdot Slp1_{\text{A}}\right]\cdot MPF$ |
| 5 | $MPF+Rum1\xrightarrow{}MPFRum1$ | $w_{5}=\frac{k_{\text{ASS}}}{\Omega}\cdot MPF\cdot Rum1$ |
| 6 | $MPFRum1\xrightarrow{}MPF+Rum1$ | $w_{6}=k_{\text{DISS}}\cdot MPFRum1$ |
| 7 | $MPFRum1\xrightarrow{}MPF$ | $w_{7}=k_{\text{DRUM1}}\cdot MPFRum1$ |
| 8 |  | $w_{8}=k_{\text{IRUM1}}\cdot MPFRum1$ |
| 9 |  | $w_{9}=\left[\frac{k_{\text{D1CYC}}}{\Omega}\cdot Slp1_{\text{T}}+\left(\frac{k_{\text{D2CYC}}-k_{\text{D1CYC}}}{\Omega}\right)\cdot Slp1_{\text{A}}\right]\cdot MPF_{\text{P}}$ |
| 10 |  | $w_{10}=\frac{k_{\text{I2RUM1}}}{\Omega}\cdot MPF_{\text{P}}\cdot Rum1$ |
| 11 | $\xrightarrow{}Rum1$ | $w_{11}=V_{\text{SRUM1}}\cdot\Omega$ |
| 12 | $Rum1_{\text{P}}\xrightarrow{}Rum1$ | $w_{12}=k_{\text{ARUM1}}\cdot Rum1_{\text{P}}$ |
| 13 | $MPFRum1\xrightarrow{}Rum1$ | $w_{13}=k_{\text{DMPFRUM1}}\cdot MPFRum1$ |
| 14 | $Rum1\xrightarrow{}$ | $w_{14}=k_{\text{DRUM1}}\cdot Rum1$ |
| 15 | $Rum1_{\text{P}}\xrightarrow{}$ | $w_{15}=\frac{k_{\text{DRUM1P}}}{\Omega}\cdot\left(MPF+\alpha\cdot MPF_{\text{P}}\right)\cdot Rum1_{\text{P}}$ |
| 16 | $Rum1_{\text{P}}\xrightarrow{}$ | $w_{16}=k_{\text{DRUM1}}\cdot Rum1_{\text{P}}$ |
| 17 | $Wee1_{\text{P}}\xrightarrow{}Wee1$ | $w_{17}=\left(V_{\text{WEE1}}\cdot\Omega\right)\cdot\frac{Wee1_{\text{P}}}{\left(J_{\text{1WEE1}}\cdot\Omega\right)+Wee1_{\text{P}}}$ |
| 18 |  | $w_{18}=k_{\text{WEE1}}\cdot\left(MPF+\alpha\cdot MPF_{\text{P}}\right)\cdot\frac{Wee1}{\left(J_{\text{2WEE1}}\cdot\Omega\right)+Wee1}$ |
| 19 |  | $w_{19}=k_{\text{CDC25}}\cdot\left(MPF+\alpha\cdot MPF_{\text{P}}\right)\cdot\frac{Cdc25}{\left(J_{\text{1CDC25}}\cdot\Omega\right)+Cdc25}$ |
| 20 | $Cdc25_{\text{P}}\xrightarrow{}Cdc25$ | $w_{20}=\left(V_{\text{2CDC25}}\cdot\Omega\right)\cdot\frac{Cdc25_{\text{P}}}{\left(J_{\text{2CDC25}}\cdot\Omega\right)+Cdc25_{\text{P}}}$ |
| 21 |  | $w_{21}=k_{\text{1SLP1}}\cdot IE_{\text{A}}\cdot\frac{Slp1}{\left(J_{\text{1SLP1}}\cdot\Omega\right)+Slp1}$ |
| 22 | $Slp1_{\text{A}}\xrightarrow{}Slp1$ | $w_{22}=\left(V_{\text{2SLP1}}\cdot\Omega\right)\cdot\frac{Slp1_{\text{A}}}{\left(J_{\text{2SLP1}}\cdot\Omega\right)+Slp1_{\text{A}}}$ |
| 23 |  | $w_{23}=k_{\text{1IE}}\cdot\left(MPF+\alpha\cdot MPF_{\text{P}}\right)\cdot\frac{IE}{\left(J_{\text{1IE}}\cdot\Omega\right)+IE}$ |
| 24 | $IE_{\text{A}}\xrightarrow{}IE$ | $w_{24}=\left(V_{\text{2IE}}\cdot\Omega\right)\cdot\frac{IE_{\text{A}}}{\left(J_{\text{2IE}}\cdot\Omega\right)+IE_{\text{A}}}$ |
| 25 | $\xrightarrow{}Mass$ | $w_{25}=\mu\cdot Mass$ |
| 26 |  | $w_{26}=k_{\text{DX}}\cdot CCP\cdot Rum1$ |
| 27 |  | $w_{27}=k_{\text{DX}}\cdot CCP\cdot MPFRum1$ |
| 28 |  | $w_{28}=k_{\text{DX}}\cdot CCP\cdot Rum1_{\text{P}}$ |
