# Supplementary material for: Cell Cycle Control by a Minimal Cdk Network
Source: PLoS Comput Biol. 2015 Feb 6;11(2):e1004056. doi: 10.1371/journal.pcbi.1004056 (PMC4319789; doi:10.1371/journal.pcbi.1004056)
Supplement: S5 Table — (DOCX) [file pcbi.1004056.s005.docx]

**Table S5. Strains used in this study**

| **Strain** | **Genotype** | **Source** |
| --- | --- | --- |
| DC147 | *h?*  *leu1Δ::Pcdc13::cdc13-L-cdc2::cdc13 3'UTR::ura4+ cdc13Δ::natMX6 ura4-D18* | This study |
| DC235 | *h+*  *leu1Δ::Pcdc13::cdc13-L-cdc2::cdc13 3'UTR::ura4+ cdc2Δ::kanMX6 cdc13Δ::natMX6 cig1Δ::ura4+ cig2Δ::ura4+ puc1Δ::ura4+ ura4-D18* | [7] |
| DC276 | *h+*  *leu1Δ::Pcdc13::cdc13-L-cdc2AF::cdc13 3'UTR::ura4+ cdc2Δ::kanMX6 cdc13Δ::natMX6 cig1Δ::ura4+ cig2Δ::ura4+ puc1Δ::ura4+ ura4-D18* | [7] |
| DC318 | *h+*  *leu1Δ::Pcdc13::cdc13-L-cdc2::cdc13 3'UTR::ura4+ cdc2Δ::kanMX6 cdc13Δ::natMX6 cig1Δ::ura4+ cig2Δ::ura4+ puc1Δ::ura4+ rum1Δ::hphMX6 ura4-D18* | [7] |
| DC358 | *h+*  *leu1Δ::Pcdc13::cdc13-L-cdc2::cdc13 3'UTR::ura4+ cdc2Δ::kanMX6 cdc13Δ::natMX6 cig1Δ::ura4+ cig2Δ::ura4+ puc1Δ::ura4+ wee1Δ::ura4+ mik1Δ::leu2 ura4-D18* | [7] |
| DC365 | *h-*  *wee1-50 mik1Δ::leu2+ leu1-32* | This  study |
| DC415 | *h+*  *cig1Δ::hphMX6 cig2Δ::kanMX6 puc1Δ::hphMX6* | This  study |
| DC416 | *h?*  *leu1Δ::Pcdc13::cdc13-L-cdc2AF::cdc13 3'UTR::ura4+ cdc13Δ::natMX6 ura4-D18* | This study |
|  |  |  |
| DC499 | *h-*  *wee1-50 mik1Δ::leu2+ cig1Δ::hphMX6 cig2Δ::kanMX6 puc1Δ::hphMX6 leu1-32* | This  study |
